# Measurement of free glucocorticoids: quantifying corticosteroid binding capacity and its variation within and among mammal and bird species

**DOI:** 10.1093/conphys/coaa057

**Published:** 2020-07-28

**Authors:** Brendan Delehanty, Gregory D Bossart, Cory Champagne, Daniel E Crocker, Kyle H Elliott, Patricia A Fair, Dorian Houser, Amy E M Newman, Rudy Boonstra

**Affiliations:** 1 Centre for the Neurobiology of Stress, Department of Biological Sciences, University of Toronto Scarborough, 1265 Military Trail, Toronto, ON M1C 1A4, Canada; 2 Georgia Aquarium, 225 Baker Street Atlanta, GA 30313, USA; 3 National Marine Mammal Foundation, 2240 Shelter Island Dr Suite 200, San Diego, CA 92106, USA; 4 Department of Biology, Sonoma State University, 1801 E. Cotati Ave., Rohnert Park, CA 94928, USA; 5 Department of Natural Resource Sciences, McGill University, Sainte Anne-de-Bellevue, Québec, H9X 3V9, Canada; 6 Department of Public Health Sciences, Medical University of South Carolina, 135 Cannon Street, Charleston, SC 29425, USA; 7 Department of Integrative Biology, University of Guelph, 50 Stone Rd E, Guelph, Ontario N1G 2W1, Canada

**Keywords:** cortisol, dialysis, free hormone hypothesis, harvester, interspecific variation, marine mammals, stress, wildlife

## Abstract

Plasma glucocorticoid (CORT) levels are one measure of stress in wildlife and give us insight into natural processes relevant to conservation issues. Many studies use total CORT concentrations to draw conclusions about animals’ stress state and response to their environment. However, the blood of tetrapods contains corticosteroid-binding globulin (CBG), which strongly binds most circulating CORT. Only free CORT (CORT not bound by CBG) leaves the circulation and exerts biological effects on CORT-sensitive tissues. Measuring free CORT concentrations provides insight to an animal’s stress response that cannot be revealed by simply measuring total CORT. To calculate free CORT concentrations in plasma or serum samples, one needs three measurements: the binding affinity of CBG for CORT (which varies by species), the total CORT concentration in the sample and the maximum corticosteroid binding capacity (MCBC) of CBG in the sample. Here, we detail the measurement of CBG binding capacity. We compare and contrast the three main methods to measure MCBC: charcoal, cell harvester and dialysis. Each is defined by the means by which free and bound CORT are separated. We weigh the relative merits and challenges of each. We conclude that sample volume, species and taxon binding specificity, and availability of equipment are the primary considerations in selecting the appropriate separation method. For most mammals, the charcoal method is recommended. For birds, the harvester method has critical advantages over the charcoal method. The dialysis method is widely regarded as the gold standard and has lower equipment costs but is more time-intensive and costly in terms of radioactive isotope needed and is less suited to processing large numbers of samples. The binding capacity of CBG varies tremendously within and among the bird and marine mammal species studied, and we discuss the implication of this variation for understanding the role of stress in wildlife.

## Introduction

The measurement of glucocorticoid levels (CORT; cortisol, corticosterone or both depending on species) are key in helping us understand vertebrate physiology (Sapolsky *et al.*, 2000), their ecology, life history and population dynamics (see the 10 review papers in the special feature on the *Ecology of Stress, Functional Ecology*: volume 27, issue 1; 2013) and their conservation (Cooke and O’Connor, 2010; Dantzer *et al.*, 2014). However, all tetrapods (amphibians, reptiles, birds and mammals) have circulating corticosteroid-binding globulin (CBG), and in virtually all, it binds CORT strongly and prevents the bound CORT from leaving the circulation (Seal and Doe, 1965; Sandberg *et al.*, 1966; Westphal, 1983; Breuner and Orchinik, 2002; Desantis *et al.*, 2013). This protein evolved to fill this role over 350 million years ago when vertebrates became terrestrial (being found in lungfish but not fish: Baker, 2002; Desantis *et al*., 2013). Evidence indicates that only free CORT in plasma (i.e. circulating hormone not bound to CBG) is able to pass out of circulation and act on CORT-sensitive tissues (Bright 1995; Qian *et al.*, 2011) (see reviews of the biomedical research by Perogamvros *et al.*, 2012; and of the wildlife research by Breuner *et al.*, 2013). Because CBG levels change within individuals as a function of sex, season, social status, reproductive condition and stress levels (Boonstra *et al.*, 2007; Edwards and Boonstra, 2017) and species vary considerably in CBG binding capacity relative to their total plasma CORT levels (Desantis *et al.*, 2013), determining free hormone levels in blood is essential for understanding the biological significance of CORT levels.

However, because all immunoassay kits for measuring CORT that we are aware of measure total CORT rather than free CORT, free CORT concentrations must be calculated. This requires determining three physiological parameters: (i) the total CORT concentration in each sample (typically measured with a CORT assay kit), (ii) the binding affinity of CBG for the CORT (this binding affinity is thought to be constant within a species at a fixed temperature but variable among species) and (iii) the maximum corticosteroid binding capacity (MCBC) of CBG in each sample. Once each of these values is determined, the free CORT concentration can be calculated using a formula based on the laws of mass action (Barsano and Baumann, 1989). We have previously published comprehensive accounts of the methods to calculate the binding affinity (represented by the equilibrium dissociation constant, *K*_d_) of CBG for a wide range of species (terrestrial mammals in Delehanty *et al*., 2015 and marine mammals in Delehanty *et al*., 2019).

Because one of the obstacles to stress researchers measuring the binding capacity of CBG may be a lack of a detailed methodology, and because there are a number of techniques to do so, we lay out in detail the three main methods and compare their efficacy for measuring MCBC in plasma or serum samples (hereinafter ‘plasma’) in mammals and birds.

The MCBC assay methods described here all involve saturating the CBG with tritium-labelled CORT ([^3^H]-CORT) and then separating the bound CORT from the free CORT. The methods are defined by the means used to accomplish this separation: the ‘charcoal method’ uses activated charcoal (Tan and Mulrow, 1975; McDonald *et al.*, 1981; Boonstra and Boag, 1992), the ‘harvester method’ uses filtration with glass-fibre filters in a cell harvester (Orchinik *et al.*, 2000) and the ‘dialysis method’ segregates bound hormones on one side of a dialysis membrane (Daughaday *et al.*, 1962; Bradley *et al.*, 1980). Although there are commercially available CBG assay kits, they are expensive and designed for use with single species (e.g. IBL America kit KIPI1809 for human and MyBioSource, Inc. kit MBS2089051 for rat) so the ability of the antibodies used in those kits to accurately measure CBG in other species would need to be validated.

Our purpose in this paper is 3-fold. First, we compare the three techniques for running MCBC assays to assess the relative merits and challenges of each. Second, based on our experience with the charcoal and harvester methods and our reading of the literature, we suspected that these techniques differed in their effectiveness partly based on the taxonomy of the study species (bird versus mammal). We therefore compared the charcoal and harvester methods in select bird and mammal species. Third, we measured the binding capacity of individuals of several species of marine mammals to investigate the degree of variability in MCBC within and among species.

## Methods

Measuring MCBC is not a single-assay procedure: separate steps are required to calculate the optimal plasma dilutions, the optimal CORT concentration required to saturated CBG, and—in the case of the harvester and charcoal methods—a correction is required to account for the loss of bound hormone during the separation procedures. To help clarify the logic of these steps, Box 1 explains the basic theory of measuring CBG binding capacity, including an explanation of how it is used in the calculation of free hormone concentrations. Box 2 includes a flow chart to illustrate the sequence of assay procedures for all three MCBC methods. The MCBC methods themselves are described fully, but succinctly, below. For those who want to measure MCBC, we have included our full laboratory protocols along with useful spreadsheets in Supporting Files 1–5.

### Buffers

The charcoal and dialysis methods use phosphate-buffered saline with gelatin (PBS; 8.66 g Na_2_HPO_4_ [anhydrous], 6.10 g NaH_2_PO_4_·2H_2_O, 1 g gelatin, diluted up to 1 L in ultrapure water and adjusted to pH 7.4 with 1 N NaOH [Sigma-Aldrich, Mississauga, Canada, Cat. Nos. S5136, 711 505, G6144 and 109137, respectively]). We used all the PBS in < 1 week, so we did not a add preservative; for longer storage, we added 0.1 g thimerosal (Sigma-Aldrich, Mississauga, Canada Cat. Nos. T5125 and S2002); sodium azide can be used as an alternative bacteriostatic agent. The harvester method uses a 50-mM Tris acetate buffer (6.05 g Trizma base [Sigma T1503] in 1 L ultrapure water, chilled to 4°C, and pH adjusted to 7.4 with 5 N acetic acid [Sigma **695092**]) for incubating plasma samples and a 25-mM Tris–HCl rinse buffer (6.05 g Trizma base dissolved in 2 L ultrapure water, chilled to 4°C, and pH adjusted to 7.4 with 6 N hydrochloric acid [Sigma **320331**]). When we refer to ‘buffer’ throughout this paper, we are referring to the appropriate assay buffer for the selected separation procedure.

### Dextran-coated charcoal

Dextran-coated charcoal (DCC) is used both to strip endogenous CORT from plasma samples and as a means of separating bound from free CORT in the charcoal separation method. DCC was prepared in one of two ways. The first method was to add 6.25 g activated charcoal (Sigma-Aldrich, Mississauga, Canada, Cat. No. C5260) and 0.625 g dextran (Sigma-Aldrich, Mississauga, Canada, Cat. No. D8821) to 100 mL ultrapure water. This was shaken vigorously until all the dextran was dissolved, then the mixture was allowed to settle for 2–4 h. The supernatant with very fine charcoal particles was decanted, while retaining the charcoal that had settled (this helps to ensure that when centrifuging the charcoal in later steps, the charcoal rapidly settles). New water was added, and the decanting process was repeated two to four times, after which the volume was returned to 100 mL and the mixture (‘DCC concentrate’) was stored at 4°C. When DCC was required in an assay, one part of DCC concentrate was added to nine parts buffer. The second method we used and came to prefer was to mix 0.05 g dextran with 100 mL buffer and stirring with a magnetic stirring bar until the dextran was fully dissolved (~20 min). We then added 0.5 g activated charcoal and stirred for another 20 min.

### Stripping plasma with DCC

Removing endogenous steroids from individual samples makes it easier to calculate the MCBC as all samples are measured with the same total concentration of [^3^H]-CORT. We removed endogenous steroids by the addition of DCC: two volumes of DCC were added to one volume of plasma in a microtube, vortexed and allowed to sit for at least 1 h at room temperature. The sample was centrifuged until the charcoal was fully pelletized at the bottom of the microtube. The supernatant (stripped and diluted one-thirds of plasma) was pipetted into a clean microtube for use in assays.

### Dilution curve to establish optimal plasma dilution

The charcoal and harvester methods depend on the ability of the charcoal or the filter to separate bound CORT from free CORT, and using very high concentrations of CBG and CORT can challenge the capacity of these separation methods. Therefore, for these methods, we use a plasma dilution assay to find an optimal plasma dilution for each species. The assay itself is best thought of as a rule of thumb: we look for a dilution that results in ~10% binding of 1 nM [^3^H]-CORT. Because the dialysis method does not have an active separation step, and based on recommendations that plasma in microdialysis should not be highly diluted, our dialysis protocol uses plasma diluted to 1/10 (i.e. one part plasma to nine parts buffer) in all cases.

To estimate the optimal plasma concentrations for the harvester and charcoal methods, we used stripped plasma pooled from up to 10 individuals. For each species, we prepared a plasma dilution series in buffer (typically final dilutions of 1/54, 1/198, 1/450 and 1/750). Plasma was incubated in 12 × 75-mm polypropylene test tubes with 1 nM [^3^H]-CORT (for total binding, TB) or 1 nM [^3^H]-CORT and 4 μM non-tritiated CORT (for nonspecific binding, NSB; see Box 1 for an explanation of TB versus NSB). Three TB replicates and two NSB replicates were run for each dilution, and the final volume in each tube was 150 μL. After incubating the tubes at 4°C for 4 h, we separated bound from unbound CORT using the charcoal or the harvester method, as appropriate.

In the charcoal method, 300 μL of ice-cold DCC was added to all tubes, vortexed and incubated in an ice-bath for 15 min before spinning in a refrigerated centrifuge set at 0°C for 12 min at 2000 × g. The supernatant, containing bound CORT, was decanted into 7-mL scintillation vials (Ultident, St. Laurent, Quebec, Cat. No. 17-S207-5), to which 2.5 mL scintillation fluid was added (any scintillation cocktail capable of handling aqueous samples is suitable, e.g. EcoLite, MP Biomedicals, Santa Ana, USA). Total counts (TOTCNT) were measured by adding the same amount of [^3^H]-CORT as was added to the plasma samples directly into three scintillation vials. Vials were vortexed and counted in a scintillation counter. Specific binding (SB; i.e. strongly bound to CBG) was calculated in counts per minute (cpm) by subtracting the NSB counts (i.e. weakly bound to albumin and other plasma components) from the TB counts (Box 1). We used this dilution curve to select a sample dilution that would result in SB cpm/TOTCNT cpm ≈ 10% to then use for subsequent MCBC assays for this species.

For the harvester method, a Whatman GF/B glass fibre filter (Biomedical Research and Development Laboratories Inc., Gaithersburg, MD, USA) was soaked in refrigerated 25 mM Tris–HCl rinse buffer with 0.3% polyethylenimine for 1 h, then placed in the cell harvester (48-channel Brandel harvester, Biomedical Research and Development Laboratories Inc., Gaithersburg, MD, USA). The test tubes were placed in the harvester apparatus, and the samples were aspirated along with three 3-mL washes of ice-cold 25 mM Tris–HCl rinse buffer. Because rubber o-rings compress the filter around the area where each channel is filtered, the filter is scored and the disk of filter paper that contains the CBG-bound [^3^H]-CORT can be removed for scintillation counting. The filter disks were removed to scintillation vials, 300 μL of 100% ethanol was added to the vial and the vials were placed on an orbital shaker at ~200 rpm for 1 h. Next, 2.5 mL scintillation fluid (Ultima Gold MV, Perkin Elmer, Groningen, Netherlands) was added to each vial; the vials were vortexed thoroughly and then placed on an orbital shaker for 1 h. The vials were then placed in a scintillation counter, and %SB was calculated the same way as for the charcoal method.

**Figure 1:**
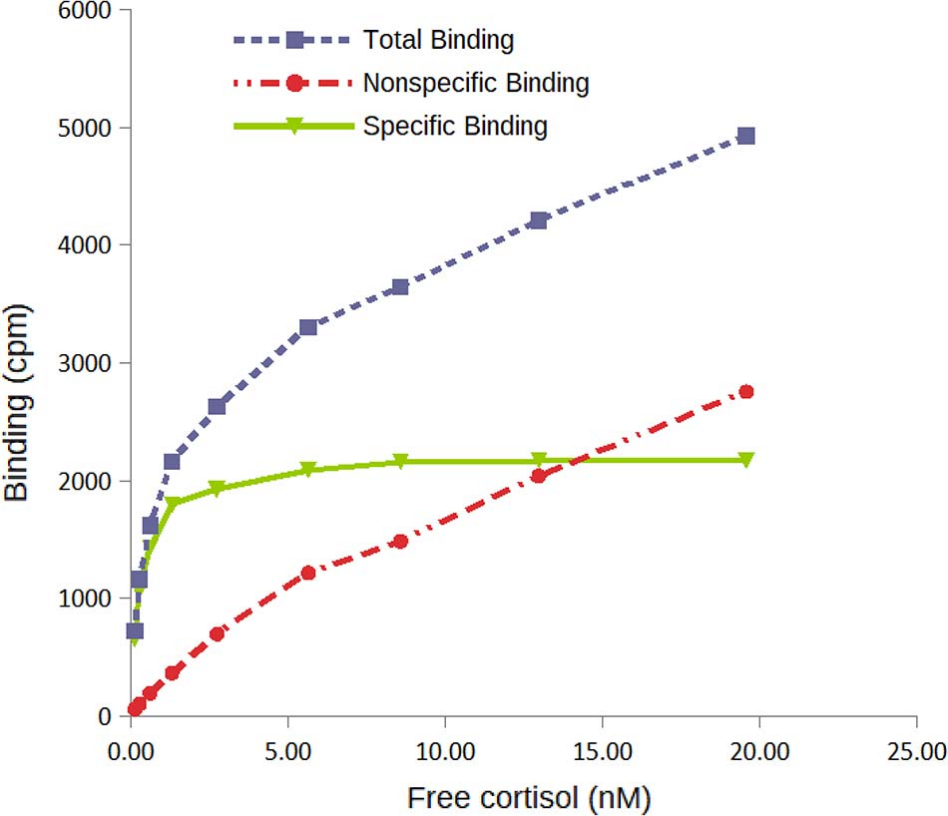
Saturation binding curve at 4°C for Antarctic fur seal (data from Delehanty *et al*., 2019). Specific binding (by CBG) is the difference between total binding and nonspecific binding. Visual inspection suggests that the asymptote is almost reached at ~5 nM free cortisol, and by 15 nM free cortisol the nonspecific binding exceeds specific binding. Using this data, we chose to add 10 nM cortisol to plasma samples in the MCBC assay for Antarctic fur seals. Note that in practice, because total and nonspecific binding counts increase steadily as cortisol concentrations increase, small pipetting errors will result in increasing errors in calculating the specific binding. Therefore, we use the point where the nonspecific binding crosses the specific binding line as a rough maximum GC concentration for the MCBC assay. This is largely an issue for the DCC (used for this curve) and dialysis methods, as the harvester method has much lower nonspecific binding counts

### Determining saturating hormone concentrations: charcoal and harvester methods

All MCBC methods require saturation of the CBG in the diluted plasma mixture by adding sufficient [^3^H]-CORT. Because CBG levels can vary substantially among individuals or among samples collected at different times of the year (e.g. Delehanty and Boonstra, 2011), the MCBC assay must add enough [^3^H]-CORT to saturate plasma from the sample with the most CBG. However, if too much [^3^H]-CORT is added, measurement error increases. We identified the optimal saturation concentration by examining the saturation binding curve that was used to determine the equilibrium dissociation constant (*K*_d_) for the species of interest (see Delehanty *et al*., 2015 and Delehanty *et al*., 2019 for details on performing saturation binding assays). The saturation binding curve is produced by incubating plasma with an increasing amount of [^3^H]-CORT. Fig. 1 is a saturation binding curve for the Antarctic fur seal (data from Delehanty *et al*., 2019). Note that the x-axis is the free hormone concentration in the plasma mixture at equilibrium, not the total CORT concentration added to the mixture: a portion of the CORT added to the plasma solution is bound to the CBG. A visual inspection of the curve suggests that the CBG was saturated starting around 5 nM free cortisol and that by 15 nM free cortisol the nonspecific binding exceeds specific binding (as nonspecific binding increases, the potential for error in calculating the specific binding increases). A concentration of 10 nM appeared to be a good compromise: high enough that samples with significantly more CBG than the pooled plasma used for running the saturation curve would still be saturated, but low enough that high total and nonspecific binding counts were unlikely to contribute to errors in calculating specific binding. To confirm how much variation in CBG levels could be accommodated, we calculated that with a *K*_d_ at 4°C of 0.26 nM, and at a 1/450 plasma dilution, the asymptote of the saturation curve was 2200 cpm. Based on the activity of the tritiated cortisol used, 2200 cpm corresponds to 0.2 nM binding capacity. Because saturation binding assays are run using plasma pools made up of several individuals, we expect that some individual samples will have higher CBG levels. If we assume that individual Antarctic fur seals could have up to 10× more CBG than the plasma pool, it would mean that some MCBC samples could have 2 nM binding capacity (in a 1/450 dilution). By using 10 nM [^3^H]-CORT, the free cortisol concentration would be 8 nM, which is still well within the approximate asymptote of the saturation curve. Saturating concentrations for all species were determined using this method.

### Determining saturating hormone concentrations: dialysis method

Using the dialysis method for measuring MCBC, we always diluted plasma to 1/10, so a higher concentration of [^3^H]-CORT was required to saturate the CBG than was required in the charcoal or harvester methods. As with the charcoal and harvester methods, we calculated the saturating CORT concentration for the dialysis method based on species’ saturation binding curves. For example, the Antarctic fur seal saturation curve in Fig. 1 has an asymptote at 2200 cpm, which corresponds to 0.2 nM given the specific activity of the [^3^H]-CORT used. That curve was generated with plasma diluted to 1/450. Therefore, a 1/10 dilution would have 9.0 nM binding capacity. Using a target of 8 nM free CORT and ensuring that samples with up to 10× higher CBG levels than the pooled plasma are also saturated, we would need to add 10 ^*^ 9 nM + 8 nM = 98 nM [^3^H]-CORT. At such high CORT concentrations, it is costly to use pure [^3^H]-CORT, so we prefer to make up the total concentration using a mixture of [^3^H]-CORT and highly purified non-labelled CORT (e.g. cortisol from Sigma-Aldrich, Oakville, Canada, Cat. No. C^−106^). For example, for 98 nM total CORT, we would use 9.8 nM [^3^H]-CORT and 88.2 nM non-labelled CORT. A spreadsheet with calculations to select dilutions and volumes of [^3^H]-CORT is available in Supporting File 2 published with the online version of this article.

**Figure 2:**
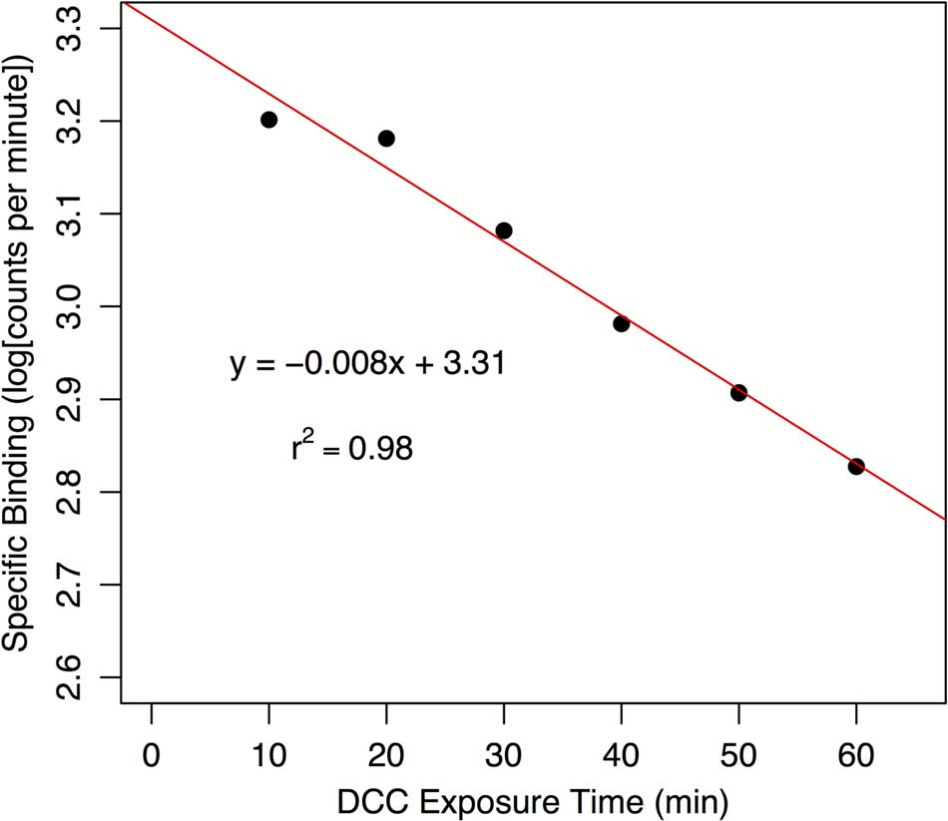
Loss of CBG-bound cortisol (measured as the logarithm of scintillation counts per minute) to dextran-coated charcoal (DCC) as a function of time in the bottlenose dolphin. The loss over 10 min exposure to DCC. Using the regression equation, the ratio of the specific binding at time 0 (*y* = 10^3.31^ = 2042 cpm) to that at the DCC exposure time of 10 min (*y* = 10^–0.008 * 10 + 3.31^ = 1698 cpm) is 1.07, which is the correction factor to be applied to the MCBC measurements using DCC. This adsorption curve should be run for each new batch of DCC

### Comparing separation methods: charcoal

As explained in Box 1, we measure total binding (TB) and nonspecific binding (NSB) and use those values to calculate specific binding (SB)—the measure of CBG binding capacity. For each sample, we prepared 3-TB tubes with stripped plasma, [^3^H]-CORT and buffer added to 12 × 75 polypropylene test tubes in quantities to result in a 150-μL reaction volume with the optimized plasma dilution and [^3^H]-CORT concentration for the species. We prepared two NSB tubes for each sample, comprised of stripped plasma, [^3^H]-CORT and buffer with unlabeled CORT added in quantities to match the concentrations in the TB tubes with the addition of a final unlabeled CORT concentration of 4 μM. The tubes were vortexed, briefly spun in a centrifuge, covered with plastic wrap and incubated in the refrigerator overnight. The next morning, we proceeded with the charcoal method of separation as described for the dilution curve assay.

For each sample, we calculated specific binding in cpm (SB_cpm_) from the TB and NSB counts (Box 1). Total count (TOTCNT) vials were prepared by pipetting directly into three scintillation vials with 3 mL scintillation fluid the amount of [^3^H]-CORT added to samples. We converted the SB_cpm_ to nM specific binding (SB_nM_) using the cpm of the total count vials (TOTCNT_cpm_) and the known amount of [^3^H]-CORT added (nM_TOTAL_) as follows: SB_nM_ = SB_cpm_/(TOTCNT_cpm_/nM_TOTAL_). Details of these calculations can be found in Supporting File 3 with the online version of this article. The SB_nM_ represents the CBG binding capacity in the diluted plasma sample, so we multiplied it by the dilution factor to arrive at the ‘unadjusted MCBC’ of undiluted plasma in nM.

This MCBC is ‘unadjusted’ because during the charcoal separation procedure the DCC adsorbs free CORT irreversibly and, as free CORT is removed from solution, the binding equilibrium will shift so that there is a net release of CBG-bound CORT. Previous research using DCC assumed that it did not significantly affect CBG-bound CORT, but here we show that this assumption is wrong. This results in an underestimate of MCBC. To correct for this loss, we ran a ‘charcoal adjustment assay’ for each species using pooled plasma. A series of TB and NSB test tubes were set up as in the MCBC assay, but using pooled plasma. The length of DCC exposure was varied (usually six intervals ranging from 5 to 60 min), and the log(SB_cpm_) was plotted as a function of incubation time (e.g. Fig. 2). The resulting regression equation was used to calculate a multiplier to correct the unadjusted MCBC for the loss of CBG-bound CORT to the charcoal (spreadsheets to perform these calculations are provided in Supporting Files 3 and 5 at *Conservation Physiology* online).

### Comparing separation methods: harvester

The MCBC assay using the harvester method begins with incubating samples in 12 × 75 polypropylene test tubes set up identically to the charcoal method (i.e. for each species, the same 150-μL reaction volume, CORT concentrations and plasma dilution as would be used in the charcoal method), but using Tris acetate buffer (see above) for the DCC used to strip plasma, for diluting the plasma and for the [^3^H]-CORT and unlabeled CORT solutions. While setting up the tubes, we added the same amount of [^3^H]-CORT as was used in the TB and NSB tubes for each species to a small piece of glass-fibre filter paper in each of three scintillation vials. These provide the TOTCNT values for converting cpm to nM binding. The separation procedure and counting of filter disks for the MCBC samples then proceeded in the same way as described for the dilution curve, above.

### Comparing separation methods: dialysis

For MCBC by the dialysis method, we used a reusable 96-well dialysis unit (HTDialysis, Gales Ferry, CT, USA) with vertical dialysis membranes dividing each well. We used Spectra/Por 2 dry membrane with a 12–14-kD MWCO (Cat. No. 132678, Spectrum Laboratories, Rancho Dominguez, CA) hydrated in accordance with HTDialysis instructions.

The solutions used in this technique differ slightly from those of the previous methods because the separation of free and bound hormone occurs across the membrane at equilibrium rather than as the result of treating a single volume of plasma and hormone (see explanation in Supporting File 1 published with the online version of this article). Each well had a ‘plasma side’ and a ‘buffer side’, both having a volume of 150 μL. The plasma side of wells had 10% plasma, and the predetermined saturating concentration of [^3^H]-CORT in buffer with (NSB wells, three replicates) or without (TB wells, four replicates) 4 μM unlabeled CORT. The buffer side of wells matched the plasma sides but did not contain plasma. The dialysis unit was refrigerated (~4°C) on an orbital shaker at ~100 rpm for ~6 h. After the equilibration period, 100 μL from the plasma side and 100 μL from the buffer side of each well were pipetted into two scintillation vials, and 2.5 mL of scintillation fluid was added to each vial. After thorough vortexing, the vials were placed in a scintillation counter. The TB and NSB cpm were calculated by subtracting the buffer side cpm from the plasma side cpm of each individual well. The SB cpm was calculated by subtracting the mean NSB cpm from the mean TB cpm. As with previous methods, the counts were converted to nM by adding the saturating concentration of [^3^H]-CORT directly to three scintillation vials and using the cpm of that known concentration to calculate the cpm/nM of [^3^H]-CORT. A complete explanation of this calculation is provided in Supporting File 1, and a spreadsheet for performing the calculations is in Supporting File 3 at the online version of this article.

### Comparing separation methods: sample selection

As part of another project (Champagne *et al*., 2018), we ran MCBC assays using a cell harvester on hundreds of individual plasma samples from bottlenose dolphins (*Tursiops truncatus*). We selected 24 of these samples covering a wide range of binding capacities (range: 9 to 73 nM by the harvester method) to assess by all three separation methods. Equilibrium dialysis is considered the gold standard method for measuring binding capacity (Zeitlinger *et al*., 2011), so we assumed that our dialysis concentrations were most accurate and compared charcoal separation and cell harvester methods against dialysis.

## Comparison of MCBC in birds and mammals

Seemingly by chance, most studies that measure MCBC in birds have used the harvester method (e.g. Orchinik *et al.*, 2000; Love, *et al.*, 2004; Elliott *et al.*, 2014) whereas mammalian studies have tended to use the dialysis or charcoal methods (e.g. Tan and Mulrow, 1975; McDonald *et al.*, 1981; Boonstra and Boag, 1992). During our attempts to measure marine mammal MCBC using the harvester method, we found that we often had notably high levels of variation between filters. The cell harvester uses a polycationic polymer, PEI, to bind the CBG passing through the filter (Orchinik *et al*., 2000). This coating is essential to the ability of the filter to capture CBG: in the absence of PEI, virtually no CBG is retained by the filter (unpublished data). Thus, we hypothesized that mammalian CBG may have less pronounced negative charges than avian CBG, making it less likely to be retained by the filter and more susceptible to variation in PEI coating of filters.

To test this hypothesis, we compared the consistency of mammalian and avian plasma MCBC measurements over four sequential filters. We used pooled plasma of four bird and four marine mammal species. The bird species were black-legged kittiwakes (*Rissa tridactyla*), common murre (*Uria aalge*), savannah sparrow (*Passerculus sandwichensis*) and turkey vulture (*Cathartes aura*); the marine mammals were bottlenose dolphin, Antarctic fur seal, Weddell seal (*Leptonychotes weddellii*) and sea otter (*Enhydra lutris*).

Because we were aware of concerns from bird researchers about the loss of bound hormone to DCC in the charcoal method, we also ran the charcoal adjustment assay on three of the bird species (turkey vultures have very little CBG, so all of our plasma was used up in the harvester comparison) alongside the same four marine mammal species. Plasma dilutions for all species were the same as for the harvester except sea otters, which were run at 1/100. To maximize consistency, all seven species were run at the same time, using the same batch of DCC.

### Survey of marine mammal binding capacities

To investigate the range of MCBC among marine mammal species and among individuals within a species, we measured binding capacities in five species of pinnipeds: Antarctic fur seal, Australian fur seal (*Arctocephalus pusillus doriferus*), crabeater seal (*Lobodon carcinophaga*), Weddell seal and California sea lion (*Zalophus californianus*). Each species had 9–30 individual samples. We used the charcoal method (including the charcoal adjustment assay) to measure MCBC for each of these species.

## Results

### Comparing separation methods

The optimal dilution of bottlenose dolphin plasma for the charcoal and harvester methods was calculated to be 1/16 (data not shown), and the loss of CBG-bound hormone to DCC for the bottlenose dolphin plasma was 6.5% over a 10-min exposure time, resulting in a charcoal adjustment factor of 1.07 (calculated from charcoal adsorption curve, Fig. 2).

**Figure 3:**
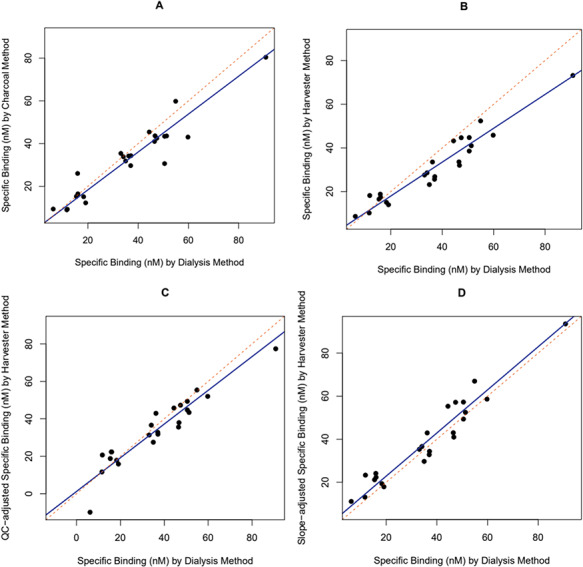
In the bottlenose dolphin plasma, the relationship between maximum corticosteroid binding capacities (MCBC) estimated by the dialysis method and: (**A**) the charcoal method corrected for loss of bound hormone to charcoal, (**B**) the harvester method with no data correction, (**C**) the harvester method with a correction based on quality control values and (**D**) the harvester method with a correction based on forcing the slope to 1

We compared MCBC methods using Deming regressions of either charcoal or harvester data against the dialysis estimates (Fig. 3). Unlike ordinary least squares regressions, the Deming method can be used when there are measurement errors in both the independent and dependent variables, making it a useful technique for comparing two assay methods (Linnet, 1993). When we compared the charcoal method to the dialysis method (Fig. 3A), the intercept was not significantly different from zero (intercept = 0.6; 95% CI: −3.5—4.7), and the slope was not significantly different from 1 (slope = 0.89; *t*_22_ = −1.82, *P* = 0.08). In the regression of harvester against dialysis data (Fig. 3B), the intercept was not different from zero (intercept = 2.5; 95% CI: −1.4—5.4), but the slope was significantly different from 1 (slope = 0.78; *t*_22_ = −5.11, *P* < 0.0001).

The binding capacity estimates for the quality control plasma used in all three methods are shown in Table 1. Because there was marked variation among the quality control values in the harvester run, we ran a second regression on the harvester data after adjusting the data from each filter by the degree to which the quality control value fell below the presumed actual specific binding of quality controls based on the mean of the dialysis and DCC values (mean = 51.3 nM). Thus, the samples on the first filter (Table 1) were all adjusted upwards by a factor of (51.3 nM/43.4 nM) = 1.18. In this regression (Fig. 3C), the intercept was significantly >0 (intercept = 5.2; 95% CI: 1.7–8.1), and the slope was still significantly different from 1 (slope = 0.81; *t*_22_ = −4.77, *P* < 0.0001).

### Comparing birds and mammals

**Figure 4:**
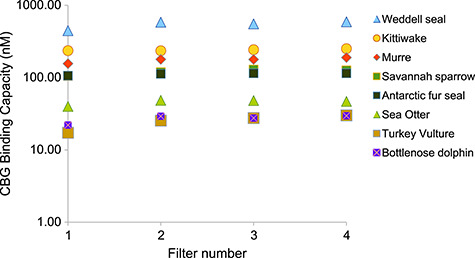
MCBC (nM) of CBG in the plasma of four bird and four marine mammal species, measured using the cell harvester method in four sequential filters. Binding capacities shown are corrected for plasma dilutions, which were as follows: kittiwake 1/100, murre 1/50, savannah sparrow 1/200, and turkey vulture 1/10, bottlenose dolphin 1/16, Antarctic fur seal and sea otter 1/200 and Weddell seal 1/1500.

When we compared the performance of the harvester on a selection of bird versus mammal plasma, results were consistent among filters for both bird and mammal species (Fig. 4); there was, however, a small but significant increase in measured MCBC from the first to fourth filters (effect of filter in repeated-measures ANOVA, *F*_3,21_ = 3.14, *P* = 0.047).

Because the turkey vulture plasma had such low CBG concentrations, we did not have enough plasma to test the loss of bound hormone to charcoal. In the remaining three bird species, the rate of loss of CBG-bound CORT to charcoal was significantly greater in birds than the mammals (Table 2; mean slope for birds was −0.022, and that for mammals was −0.0093; *t*_5_ = −3.9, *P* = 0.01). When we added the loss rate data from different batches of DCC that were run for the survey of marine mammal binding capacities (averaging the results for Antarctic fur seals and Weddell seals that were included in both datasets), there remained a significant loss of CBG-bound CORT to the DCC (Table 2; *t*_5_ = −5.6, *P* < 0.001). We also had data from previous work for the deer mouse, *Peromyscus maniculatus* (for which corticosterone is the dominant CORT). The rate of loss calculated at that time was within the range of the other mammal species (Table 2).

### Survey of marine mammal binding capacities

**Figure 5:**
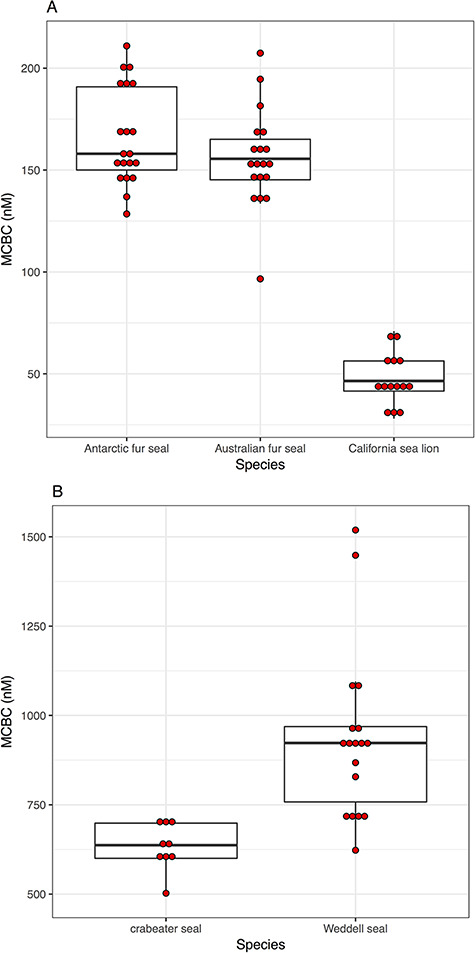
Box plots showing the maximum corticosteroid binding estimates for five species of marine mammals with low (panel **A**) and high (panel **B**) maximum corticosteroid binding capacities (MCBC)

The MCBC values of five pinniped species are presented in Fig. 5. The MCBC concentrations vary markedly within this clade. Crabeater and Weddell seals, both phocid species, had MCBC concentrations 4× – 6× that of Antarctic and Australian fur seals and 13× – 19× that of California sea lions (all three of which are otariids). Even within a species, there is a 1- to 2-fold difference between the lowest and highest values, though the California sea lions and the crabeater seals are tightly clustered.

## Discussion

### Comparing separation methods

Once the charcoal method results were corrected for the loss of CBG-bound hormone to charcoal, the estimates of MCBC were very similar to those obtained by dialysis. In the regression of charcoal and dialysis data, the intercept was not significantly different from 0; the slope was not significantly different from 1. These suggest that dialysis and charcoal yield very similar results as long as the charcoal MCBC values are corrected for the loss of CBG-bound hormone to charcoal during the DCC exposure period. In contrast, the harvester method consistently yielded lower MCBC values than those measured by dialysis, apparently due to a variable proportion of CBG passing through the filter. The regression slope of dialysis and harvester binding capacities was significantly different from 1, and harvester-derived MCBC values need to be adjusted upward by 28% to estimate the ‘true’ value as determined by dialysis (i.e. the filters are missing 22% of total CBG). The binding capacity of the quality control plasma was similar in the dialysis and charcoal runs (mean of 51.5 nM in the two dialysis runs, 51.1 nM in the single charcoal run) whereas the four harvester runs ranged from 40.2 to 48.6 nM (Table 1). The harvester data were part of a larger project that involved 41 separate filters, all of which used the same pooled plasma for the quality control samples. The inter-assay coefficient of variation for these runs (unpublished data) was 13.6% and the binding capacity ranged from 29.7 to 50.8 nM, with a mean of 38.6 nM. This suggests to us that (i) the harvester underestimates binding capacity, probably because it is failing to capture CBG, and (ii) that the proportion of CBG captured by the filters can vary markedly between runs.

**Table 1:** Estimated binding capacities of pooled bottlenose dolphin *(Tursiops truncatus*) plasma used in all runs of all separation methods

Method:	Harvester	Dialysis	Charcoal
Run 1	43.4 nM	50.5 nM	51.1 nM
Run 2	45.3 nM	52.4 nM	
Run 3	40.2 nM		
Run 4	48.6 nM		
Mean	44.4 nM	51.5 nM	n/a

There are two ways to try to correct for these shortcomings of the harvester method. First, one can measure the true MCBC of the quality control plasma (e.g. by dialysis) and then adjust each filter by the degree to which the quality control plasma falls below the true value. When we did this (Fig. 3C), the slope of the regression was still significantly different from 1, meaning that this attempted correction was not successful with these data. The second way to correct the harvester data is to measure a subset of data with dialysis or the charcoal method and use the regression equation to correct the harvester data. When we did this (Fig. 3D; all harvester MCBC values multiplied by 1.28), the intercept was not significantly different from 0 (intercept = 2.8; 95% CI = −0.20–2.2). This means that just multiplying the harvester data by the factor which required the slope of the regression equation is the best method of correcting harvester data to obtain reliable binding capacities.

**Table 2:** To examine the possible effect of taxonomic grouping on the rate of loss of CBG-bound glucocorticoids (cortisol or corticosterone) to dextran-coated charcoal (DCC) during the charcoal separation method, we ran the charcoal adjustment assay (see Methods) in selected bird and mammal species

		Slope	% loss at 15 min	Glucocorticoid used
Single batch of DCC	Murre	−0.019	49%	Corticosterone
	Kittiwake	−0.029	63%	Corticosterone
	Savannah sparrow	−0.018	47%	Corticosterone
	Bottlenose dolphin	−0.012	35%	Cortisol
	Weddell seal	−0.009	26%	Cortisol
	Antarctic fur seal	−0.006	20%	Cortisol
	Sea otter	−0.010	29%	Cortisol
Separate batches of DCC	Deer mouse	−0.011	32%	Corticosterone
	Antarctic fur seal	−0.008	24%	Cortisol
	Weddell seal	−0.011	32%	Cortisol
	California sea lion	−0.007	23%	Cortisol
	Crabeater seal	−0.013	37%	Cortisol
	Australian fur seal	−0.008	23%	Cortisol

All species in the ‘single batch’ rows were run at the same time, using the same DCC. The species in the ‘separate batch’ rows were done in advance of measuring the MCBC for the survey of marine mammal binding capacities, except the deer mouse (Peromyscus maniculatus) which was from previous unpublished data. Rate of loss was defined as the slope of a linear regression of the log (specific binding) in counts per minute versus length of DCC exposure (minutes). All slopes were significantly different from 0 (P < 0.01)

### Comparing birds and mammals

We did not initially set out to compare how separation procedures may be influenced by taxonomic group, so our data are limited in scope. The relatively high degree of variation in bottlenose dolphin MCBC of quality control samples run through the harvester led us to suspect that the harvester was highly variable in capturing CBG in our mammalian samples—something that has not, to our knowledge, been a concern in the bird studies that have used the harvester. However, when we compared the MCBC of four bird and four mammal species over four filters, it was clear that where technique and assay conditions are kept uniform, the harvester yields consistent results for both bird and mammals. Although the harvester method did show a significant increase in measured MCBC over the course of the four filters for both birds and mammals, the overall effect was very small and is of less concern than the fact that the harvester is failing to capture 22% of CBG in bottlenose dolphin plasma.

There does, however, appear to be a difference between birds and mammals in the rate at which CBG-bound CORT dissociates from the CBG and is adsorbed by the DCC during the charcoal separation method. Faster rates of loss make MCBC estimates by the charcoal method less precise. As the rate of loss increases, slight differences in DCC exposure time (e.g. from first to last sample when pipetting DCC in a single run, or slight differences between runs) have a larger impact on MCBC values. The three bird species lost CBG-bound corticosterone to DCC faster than the mammals lost CBG-bound cortisol to DCC, raising the possibility that the reason for the difference lies in either the nature of the steroid (corticosterone versus cortisol) or the taxonomic grouping (birds versus mammals). However, in previous unpublished work, we calculated the rate of loss of bound corticosterone to DCC in the deer mouse (Table 2) and we found that the corticosterone was lost to charcoal in deer mouse plasma at a rate similar to the mammals in this study. This was a small sample, but if this pattern holds true more generally, then it suggests that the charcoal method will be more challenging for bird researchers than those studying mammals.

Two other issues could potentially complicate the measurement of CBG. First, sex hormone binding globulin (SHBG) could be binding some of the CORT because stripping with DCC removes the sex hormones that would normally bind preferentially to the SHBG. The issue is restricted to mammals, as birds do not have SHBG (Wingfield *et al*., 1984; Deviche *et al*., 2001; Charlier *et al*., 2009). In mammals, the evidence suggests that SHBG binding of CORT contributes very minimally to CBG-binding estimates (Hammond and Lähteenmäki, 1983). Second, some mammals have both cortisol and corticosterone (birds have only the corticosterone). Trying to tease out the relative contributions of each under stress and their binding to CBG complicates matters. In general, where they both occur, cortisol is the dominant one and only it has the major impact on bodily processes (e.g. Kass *et al*., 1955) and responds dramatically to stress (Kastner *et al*., 1977; Bradley 1987; Boswell *et al*., 1994; Boonstra *et al*., 2001; Vera *et al*., 2011). However, the pattern is not uniform among mammals. For example, in male yellow-pine chipmunks (*Neotamias amoenus*), cortisol is 25–50 times more abundant than corticosterone, but both increase dramatically with stress (Kenagy and Place, 2000). In the European rabbits (*Oryctolagus cuniculus*), corticosterone is 11 times more abundant than cortisol, but both increase dramatically with stress (Boonstra and Tinnikov, 1998). So the presence of these two steroids in the body may need to be determined by RIA, their response to stressors and to binding by CBG by experiments and their impact by measurement. To our knowledge, this has only been done by Bradley (1987) on small insectivorous marsupial, *Phascogale calura.* Dialysis could be used to quantify the strength of binding to CBG, but the evidence indicates that corticosterone, where present, usually plays a secondary role to cortisol.

### Survey of marine mammal binding capacities

There is considerable variation in CBG concentration across seal species and among individuals. There is a 20-fold difference between the species with the most CBG (Weddell seals) and those with the least (California sea lions). Individual variation in CBG within species ranged from 1.4- and 2.5-fold differences. This variability in a small subset of marine mammals is similar to that observed in other taxa (e.g. Desantis *et al*., 2013; Delehanty *et al*., 2015). The interspecific variation is dramatic, but there are as yet no hypotheses that might explain this variation, and this would be a fertile ground for further research.

## Conclusion

The dialysis method is often considered the gold standard of separation methods (Zeitlinger *et al*., 2011) because the separation of bound and free hormone (more precisely the separation of bound from 50% of the free hormone) occurs across a semipermeable membrane at equilibrium. In contrast, charcoal separates bound from free in a single reaction volume over 10 or 15 min, during which the bound hormone is freed and progressively lost to the charcoal. With the harvester method, although the separation process is quick, there appears to be a significant portion of CBG that is not trapped by the PEI-infused filters. Thus, both charcoal and harvester methods underestimate CBG binding capacity in the absence of adjustments based on additional assays. All else being equal, dialysis would be our preferred separation method. However, the dialysis method has significant disadvantages. It was, by far, the most time and labour-intensive method. Even with a reusable 96-well dialysis plate, we found it difficult to process large sample sizes. Our standard protocol used four TB and two NSB wells per sample to allow sufficient replicates in the case of well-leakage (well contents sometimes drain from the wells in the HTDialysis plates we use, especially if the plates incubates for > 6 h). Thus, we processed only 15 samples plus 1 quality control per plate. Moreover, every well requires two scintillation tubes: the plasma side and buffer side to make a single measurement (i.e. double the number of scintillation tubes and fluid of the other methods, meaning greater expense and radioactive waste disposal). The dialysis method also used higher plasma volumes because samples are not diluted as much as with the other separation methods. This could be problematic for small species from which only small blood volumes can be collected. Using higher dilutions in the dialysis method could be possible, though it may slow the time required for the wells to reach an equilibrium, increasing the problem of well leakage.

In studies with large sample sizes in which case high throughput is important, we prefer the charcoal method. The charcoal method uses minimal plasma volumes; it is feasible to do large runs of about 90 samples per day, and the volume of radioactive waste is low relative to the other methods. The main cost of the assay is a refrigerated centrifuge; after that, the equipment and consumable costs are minimal. One drawback of the charcoal method is that it requires the extra step of running the charcoal adjustment assay (which we ran for every new batch of DCC). However, this assay uses the same equipment and techniques as the MCBC assay, which resulted in satisfactory agreement with the dialysis technique, and preparing large batches of DCC minimizes the need to repeat the charcoal adjustment assay too frequently. Thus, even though this additional step is required, it is not particularly onerous.

The harvester method involves the significant cost of the harvester (a more specialized piece of equipment than a centrifuge, so less likely to be already available or adaptable for other purposes) and creates a large volume of liquid radioactive waste. A single 48-channel filter (eight samples and one quality control) creates ~500 mL of liquid waste, albeit very low activity. If, as in our case, a researcher’s institution prohibits any drain disposal of tritium, the cost of waste disposal can be significant. Another drawback of the harvester method is the need to account for the loss of CBG through the filter. Unlike the charcoal method for which we could use another charcoal-based method to correct for the loss of CBG-bound CORT, the only way we could estimate the loss of CBG through the harvester filters was to use the dialysis method to arrive at a correction factor. Finally, using a 48-channel harvester restricted us to 8 samples plus 1 quality control per filter, which limited us to processing about 48 samples per day as we needed to prepare additional samples for the following day.

We therefore recommend the charcoal method to researchers wanting to measure CBG binding capacity, with one important caveat. The bird species we tested lost CBG-bound corticosterone to the DCC at a higher rate than what we generally see in mammals. As the rate of loss increases, slight variations in DCC exposure times will increase the MCBC measurement error. This problem is not necessarily restricted to birds—we have, on rare occasions, been unable to run saturation binding curves to measure the *K*_d_ of CBG in some mammalian species (e.g. northern elephant seals; see also Delehanty *et al*., 2015), and we have attributed this to extremely rapid loss of CBG-bound CORT to the DCC. Thus, for birds and perhaps for the occasional mammal, the harvester method may be more suitable unless the charcoal method can be further refined to measure MCBC in these species.

## Funding

This work was supported by the Office of Naval Research (ONR grant N000141512214) to R.B. Serum from some species was collected under additional ONR Grants N000141110436 to D.H. and N000141310770 to C.C. and D.H., ONR Grants N0001411IP20081 and N00014110541 to P.F. and G.B. who were also supported by the Georgia Aquarium, the Florida Protect Wild Dolphins specialty license plate program and the NOAA Fisheries Marine Mammal Health and Stranding Response Program.

## Supplementary Material

suppl_data_coaa057

## Supplementary material

Supplementary material information and files, including laboratory protocols and data analysis templates, are available at *Conservation Physiology* online.

## Box 1

### Dilutions and Saturating Concentrations

Steps 1 and 2 in the diagram above gloss over two critical questions: how dilute should the plasma be and how much [^3^H]-CORT needs to be added to saturate the CBG? Plasma is diluted in order to minimize the plasma used (more important for small species), to minimize the amount of [^3^H]-CORT used, and to minimize measurement errors when CBG and hormone concentrations are high. Using the optimal dilutions is most important for the charcoal and harvester methods because their separation processes do not perform as well at very high [^3^H]-CORT concentrations. In the Methods, we describe the dilution curve assay for estimating the optimal plasma concentration for the harvester and charcoal methods.

The dialysis method, in contrast, can work better with higher [^3^H]-CORT concentrations and with more concentrated plasma. The speed at which equilibrium is reached across a membrane is directly proportional to the ligand concentration, so using dilute plasma and low [^3^H]-CORT concentrations will require longer incubation times. Because the dialysis plates we used had a tendency for some wells to leak if left for an extended time, we used 1/10 dilution for all species.

As for calculating the saturating concentration of [^3^H]-CORT, here again there are trade-offs between using high CORT concentrations to ensure the CBG is saturated and increasing risk of measurement error that can occur when very high [^3^H]-CORT are used. As explained in the Methods and Supporting File 1, the best way to determine an optimal saturating concentration of [^3^H]-CORT is to incubate diluted plasma with increasing concentrations of [^3^H]-CORT to determine at what point specific binding is maximized. Fortunately, this is exactly what is done in a saturation binding curve that is used to determine the binding affinity (K_d_) of CBG (see Delehanty *et al*., 2015; Delehanty *et al*., 2019 for that assay, and see below to see how K_d_ fits into the calculation of free hormone. We explain in the Methods section how a saturation binding curve can be used to determine the saturating [^3^H]-CORT concentration.

### Accounting for Nonspecific Binding

In the diagram above, we have omitted an important feature of plasma: the presence of binding by plasma components other than CBG. When plasma is incubated with CORT, the bound fraction includes CORT bound by both CBG and these other plasma components (Tait & Burstein, 1964; Westphal, 1969). In particular albumin — the most abundant protein in blood —also binds CORT but does so with high capacity but low specificity and affinity (Westphal, 1969; Peters, 1995). When [^3^H]-CORT is added to plasma, the bound fraction consists of both strongly bound specific binding (SB) by CBG and weakly bound nonspecific binding (NSB). To separate out NSB, we measure binding twice. First, total binding (TB; both specific and nonspecific) is measured by adding enough [^3^H]-CORT to saturate the CBG in the plasma sample (NSB is essentially nonsaturable at concentrations used in these assays). Second, NSB is measured by adding excess unlabeled CORT in addition to the [^3^H]-CORT, so that only the NSB proteins are likely to bind any of the [^3^H]-CORT. By subtracting the NSB counts from the TB counts for each plasma sample, one can calculate the amount of specific binding by CBG. The difference between TB and NSB tubes can be visualized like this:

### Separation Methods

An essential component of measuring TB and NSB, is the ability to separate bound and free hormone. There are numerous methods available for this, but the charcoal, harvester and dialysis methods are the most common in the current literature. The charcoal method separates bound from free hormone by adding activated charcoal to the plasma mixture; the charcoal adsorbs free hormone but bound hormone remains attached to CBG or NSB components in the aqueous phase. The mixture is then centrifuged so that the free hormone is sequestered in a charcoal pellet and the bound hormone in the supernatant. The CBG-[^3^H]-CORT complex from the supernatant is then detected by liquid scintillation. For illustration purposes, we have omitted NSB:

The harvester method involves drawing the plasma–GC mixture through a glass-fiber filter that has been treated with polyethylenimine (PEI), a polycationic polymer that has the effect of binding CBG–GC complexes as they are drawn through the filter, but which allows the free hormone to pass through. The glass filters, containing the trapped CBG-[^3^H]-CORT complexes, are then placed in scintillation vials and treated so that the [^3^H]-CORT is released into the scintillation fluid to be counted.

The dialysis method uses semi-permeable membrane to separate a plasma–GC solution from a buffer solution. Because the pores in the dialysis membrane are smaller than CBG and albumin (both in the range of 50–466 kDa; Hammond et al., 1991; Peters, 1995) but large enough to let CORT molecules through, the bound hormone stays on the plasma side, whereas the free hormone concentration equilizes across the membrane. Once the system has reached equilibrium, a portion of the plasma side and the buffer side can be pipetted into separate scintillation vials and the amount of binding can be calculated by subtracting the buffer-side counts (free hormone only) from the plasma-side counts (free hormone plus bound hormone; Banker & Clark, 2008).

### Calculating the CBG Concentration

Once specific binding is calculated in counts per minute by subtracting NSB counts from the TB counts, the specific binding counts; can be converted into nM of specific binding capacity by counting a known concentration of [^3^H]-CORT in a scintillation counter to arrive at the cpm/nM of CORT. For example:

If TB = 4267 cpm and NSB = 1334 cpm, then SB = TB – NSB = 2933 cpm If the batch of [^3^H]-CORT yield 10376 cpm/nM, then SB = 2933 cpm/(10376 cpm/nM) Therefore the MCBC of the diluted sample is 0.28 nM

In the case where bound and free hormone is separated by dialysis, the nM of specific binding is equal to the concentration of CBG present in the diluted plasma sample. In the case of the charcoal method and the harvester method, one additional step for each is required (see Box 2 for a flowchart illustrating these steps).

For the charcoal separation method, a correction must be made because the nature of charcoal separation will have a tendency to underestimate binding. When charcoal is added to a plasma mixture and starts to remove the free CORT, the equilibrium between the free and bound CORT is disturbed. As the free CORT is removed, there is a net release of CBG-bound CORT. This means that some of the CBG-bound CORT present at the start of the charcoal exposure will come of the CBG and be adsorbed by the charcoal. Therefore, we need to calculate the proportion of CBG-bound CORT lost to charcoal during the separation process. We describe this “charcoal adjustment assay” in the Methods and Supporting File 1.

For the harvester method, the need to correct SB calculations is based on our observations and inferences and is not, to our knowledge, discussed elsewhere in the literature. We observe in this research and our unreported observations that the harvester underestimates CBG binding compared to the dialysis method and there can be quite high variation in binding estimates from one filter to another. We also know from unreported data that in the absence of the PEI coating, the filters retain virtually no CBG. Therefore, we infer that the harvester method is undercounting CBG binding because some CBG molecules are not trapped by the PEI and pass through the filter. We also surmise that the exact length of time a filter is soaked in the PEI and the volume and speed at which the rinse buffer is drawn through the filter can affect the proportion of CBG captured by the filters. Although we can minimize variation between filters by being as consistent as possible with filtering techniques, the only way we have found to estimate the loss of CBG through the filter is to calibrate the filter results with the dialysis method. In practice, this means that when using the harvester method to measure MCBC, one would need to select a portion of the samples (covering the range of MCBC estimates) to re-run using the dialysis method. These data could then be used to apply a correction factor to the harvester MCBC estimates (see Methods).

### Calculating Circulating Free Hormone Concentrations

Typically we measure the MCBC in order to calculate how much of circulating CORT is free. The most commonly used formula for calculating free CORT is that of Barsano & Baumann (1989), which requires knowing three pieces of information:

These values are then incorporated into the Barsano and Baumann equation, which can be expressed in simplified form as

$$ {H}_{free}=0.5\times \left[\left({H}_{total}- MCBC-{K}_d\right)+\sqrt{{\left( MCBC-{H}_{total}+{K}_a\right)}^2+\left({H}_{total}\times{K}_d\right)}\right], $$

where H_free_ is the non-CBG-bound plasma CORT in nM, H_total_ is the total plasma CORT in nM, MCBC is the nM binding capacity of CBG in the sample and the K_d_ is species-specific binding affinity (in nM) of CBG for CORT at 37°C (or appropriate body temperature).

## Box 2

Overview of the major steps and assays involved in measuring MCBC.
