# Supplementary material for: Measurement of free glucocorticoids: quantifying corticosteroid binding capacity and its variation within and among mammal and bird species
Source: Conserv Physiol. 2020 Jul 28;8(1):coaa057. doi: 10.1093/conphys/coaa057 (PMC7476546; doi:10.1093/conphys/coaa057)
Supplement: suppl_data_coaa057 [file suppl_data_coaa057.zip › Supplementary File 1 - Lab Protocols.docx]

Supplement to: “Measurement of Free Glucocorticoids: quantifying corticosteroid binding capacity and its variation within and among mammal and bird species”

## SUPPORTING FILE 1

## Laboratory Protocols for Measuring CBG Binding Capacity

NOTE: These are laboratory protocols from the Boonstra Lab at the University of Toronto Scarborough. They are intended to be useful to researchers wanting to perform assays to determine the maximum corticosteroid binding capacity (MCBC) of corticosteroid binding globulin (CBG) for cortisol or corticosterone. The commentary included in these protocols are based on our laboratory experiences and are not part of the peer reviewed paper.

## Background Information

These protocols provide the most important steps in determining the maximum corticosteroid capacity (MCBC) of CBG. These values, along with the equilibrium dissociation constant (K_d_) and the total glucocorticoid (GC) concentrations determined separately, enable you to calculate the amount of free and CBG-bound GC in plasma or serum samples.

There are several methods for measuring MCBC. The assays are very similar except for the method of separating bound and free hormone – an essential step in all cases. The present protocols address three main methods: dextran-coated charcoal (DCC) separation (the “charcoal method”) and glass fiber filtration using a cell harvester (the “harvester method”), and a microdialysis plate (the “dialysis method”).

Throughout these protocols, we attempt to keep the terminology general so that it can apply to either separation method. “Assay buffer” or just “buffer” refers to 50 mM Tris acetate if using a cell harvester to separate bound and free hormone, or phosphate buffered saline (PBS) with 0.1% gelatin (PBSg) if using DCC or microdialysis to separate.

“Undiluted Source” refers to ^3^H-CORT at the concentration shipped (1uCi/µL) whereas “Diluted source” refers to ^3^H-CORT that has been diluted 1/20 in ethanol (e.g. 250 µL undiluted source added to 4.75 mL redistilled ethanol). (“Hot” hormone refers to tritium-labeled hormone, whereas “cold” CORT refers to unlabeled hormone.)

Most species have a dominant GC – either cortisol or corticosterone. These assays can be used for either GC, so we refer simply to “CORT.” However, at times we show calculations (e.g. how much 1 mg/mL cold CORT to add to buffer to make a 12 µM solution). These calculations are all based on the molecular mass of CORTISOL users running assays with corticosterone will need to make adjustments. All calculations involving hot CORT are dependent not only on the molecular weight of the GC, but also the activity of the particular batch. Activity of tritiated CORT can range from 70-99 Ci/mmol, and the exact activity has a significant effect on volume needed to produce the desired concentration of hot CORT.

The recipes for all reagents can be found in the appendix at the end of the protocols.

IMPORTANT ABBREVIATIONS:

CBG = corticosteroid binding globulin

CORT = cortisol or corticosterone

DCC = dextran coated charcoal

NSB = nonspecific binding (binding by proteins other than CBG)

SB = specific binding (binding by CBG)

TB = total binding (combined specific and nonspecific binding)

TOTCNT = total counts (scintillation counts of known concentration of of ^3^H-CORT that are used to calculate the nM concentration of TB, SB and NSB counts)

## 1. Plasma Dilution Curve Protocol

This protocol is used to determine the optimal plasma dilution for the MCBC assay using the charcoal and harvester methods. It is meant as a first approximation and one cannot apply the results blindly. If subsequent assays are not providing optimal data at the selected dilution, it is important to adjust the plasma dilution or other factors as may be indicated.

The dialysis methods has slightly different considerations. See the dialysis discussion at the end of this section.

1. Strip and Dilute Pooled Plasma

– Prepare pooled plasma from individual plasma or serum samples in a 1.5 mL microtube. If possible, make up at least 1 mL.

– Add enough activated charcoal to the microtube to make a dark grey solution when vortexed.

– Let plasma sit at room temperature for about 1 h, vortexing occasionally to resuspend the charcoal.

– Spin down plasma mix in a centrifuge and transfer supernatant to a new microtube. You will likely need to repeat at least once to remove all the charcoal. 1 mL of raw plasma should yield about 800 µL of stripped plasma. This will be enough for the dilution curve and several saturation binding curves in most cases.

– You will need 48 µL of stripped plasma for a standard dilution curve. Once the dilution curve is prepared, freeze the remaining stripped plasma pool at -80ºC in several aliquots (to reduce freeze/thaw cycles).

– Prepare five 1.5 mL microtubes with the following dilutions (remember that plasma will be diluted by 3 in assay, so these are prepared as 3X dilutions)

| Final dilution | Dilute stripped plasma in this step by: | Take this much buffer (µL) | Add this much stripped plasma  (µL) |
| --- | --- | --- | --- |
| 1/54 | 1/18 | 340 | 20 |
| 1/100 | 1/33.3 | 388 | 12 |
| 1/198 | 1/66 | 394 | 6 |
| 1/450 | 1/150 | 894 | 6 |
| 1/750 | 1/250 | 996 | 4 |

1. Prepare ^3^H-CORT

Note that the volume of hot CORT required will depend on the specific activity of the particular lot. We sometimes use diluted hot CORT when preparing low CORT concentrations as in the dilution protocol. Therefore, when receiving a shipment of hot CORT prepare 1/20 “diluted hot CORT.” E.g. for 1 mL hot CORT, take 250 µL and add 4.75 mL ethanol to use as the diluted hot CORT.

– The dilution curve is done with a 1 nM final concentration, so make up stock at 3 nM.

•(6 tubes/plasma concentration) * (5 plasma concentrations) = 30 tubes * 50 µL/tube = 1500 µL

•make an additional 100 µL for 2 Total Counts tubes

• Total volume of 6 nM stock required =1600 µL (make 1800 µL)

For example:

At 92.9 Ci/mmol use 10 µL of DILUTED source hot CORT (i.e. hot CORT that has been diluted 1/20 from the CORT as shipped) put into 1790 µL buffer.

1. Cold CORT in buffer

--For 4 µM final concentration, prepare 12 µM stock. This is meant to be a great excess of cold CORT, so the exact concentration is not critical.

Making 12 uM stock: This assay requires 3 NSB per plasma conc. * 5 plasma conc. * 50 µL per tube = 1050 µL cold CORT. Make enough for 4 runs, or about 5 mL:

Therefore, add 22 µL of 1 mg/mL cold CORT in EtOH to 5 mL buffer

1. Set up assay

--Set up in 12 x 75 test tubes. Each plasma dilution will have 6 tubes: 3 total binding tubes (TB), 3 non-specific binding tubes (NSB)

--Add reagents in the following order using a repeating pipetter (2.5 ml tip):

1. Add 50 µL assay buffer to TB tubes ; 50 µL cold CORT in buffer to all NSB tubes.
2. Add 50 µL ^3^H-CORT in buffer to all tubes
3. Add 50 µL ^3^H-CORT to each of 2 scintillation vials. These are your total counts tubes (TOTCNT)
4. 50 µL diluted plasma to all tubes (6 tubes for each concentration of plasma)

--centrifuge tubes briefly to get all liquid to the bottom of the tubes.

--incubate for 4 h in the fridge at 4ºC

--add scintillant to TOTCNT tubes, vortex, and set aside to count with assay

5. Prepare for Separation

Harvester Separation: Soak filters in rinse buffer + 0.3% PEI in refrigerator (GF/B filters) for 1 hour prior to harvesting

Charcoal Separation: Prepare DCC and ice baths.

6. Separate

Separate according to procedures for DCC, harvester, or dialysis (procedures set out in Section 3, below).

7. Data

Enter data into the “Plasma Dilution Template” spreadsheet (Supporting File 3). Use the TB, NSB and TOTCNT values to calculate the %SB for each dilution (i.e. % Specific Binding = [TB-NSB]/TOTCNT). Choose the dilution that is closest to 10%. The idea is that you want there to be enough CBG to give a good signal, but you do not want ligand depletion.

NOTE: Running the dilution curve is not a guarantee that you have found the perfect dilution for all your future assays. Saturation binding curves still require that you use judgment in order to get the best data possible.

Note on Plasma Dilutions for the Dialysis Method:

The harvester and charcoal methods work best with relatively dilute plasma. In contrast, the dialysis method is more efficient with higher plasma concentrations (which will necessitate higher CORT concentrations to saturate the CBG, as well). Plasma dilutions of 1/10 or 1/20 are common. It is probable that dialysis can work with more dilute concentrations (especially given the wide range of CBG concentrations among species), but for any species it is advisable to test dilutions. We have limited experience with running MCBC assays with dialysis, so these are only suggestions based on our annecdotal experience.

We have found that as the plasma dilution increases, the variance between wells eventually gets too high. There is no obvious theoretical reason for this. One possibility is that because the microplate wells have a very small volume, the effect of interactions between proteins (CBG) and the walls of the wells or the dialysis membrane end up confounding results. Using higher plasma concentrations, perhaps those effects are swamped out. This is speculation, but the fact remains that dialysis will typically need to be run at higher plasma concentrations than the other methods. That being said, at high plasma concentrations, one also runs the risk of volume shift: the high protein concentration on the plasma side of the well draws in water from the buffer side. This effect can be reduced by keeping equilibration times to the minimum required to reach equilibrium (determine this by testing) and if volume shift seems to be an issue, consider decreasing the plasma concentration or increasing the protein concentration of the buffer side by adding protein (e.g. extra gelatin in the buffer-side PBS or try using dextran).

To test dilutions, try running several plasma dilutions with a saturating concentration of ^3^H-CORT, and have enough replicate wells at each plasma dilution to be able sample several (e.g., 4) wells at various times (say from 1 to 5 hours). Test how much variance is there between wells sampled at the same time, and observe how long is required for the plasma and buffer sides to reach equilibrium. Also keep an eye out for volume shift. Use this data to select a plasma dilution.

# 2. Maximum Corticosteroid Binding Capacity (MCBC) Protocol

This assay involves incubating diluted plasma in enough ^3^H-CORT to saturate the CBG. For two of the methods described here, the bound and unbound ^3^H-CORT is separated (by either charcoal or a glass-fiber filter – harvester – methods) so the bound ^3^H-CORT can be measured. For the dialysis method, a separation step is not required.

2.1 MCBC by Charcoal and Harvester Methods

A) Strip and Dilute Plasma

When using the Charcoal and Harvester methods, the MCBC assay is run with plasma diluted to the optimal dilution for that species (from the Plasma Dilution Curve protocol). Because the final step in the protocol results in a 1/3 dilution, plasma is made up here to 3X the final concentration for all MCBC methods.

To remove endogenous steroids, each plasma sample is first stripped in an initial 1/3 dilution with DCC in buffer (i.e. 1 part plasma and 2 parts DCC). This is followed by a second dilution that brings the plasma to 3X final concentration.

Each individual sample will have 3 Total Binding (TB) and 2 Nonspecific Binding (NSB) tubes, with 50 uL of 3X plasma (i.e. 250 uL without margin of safety).

Use this chart to figure out how much plasma to strip for each sample (note: the final volumes differ for the various dilutions so that the volumes of plasma are whole numbers):

| Final Dilution | 1/54 | 1/100 | 1/198 | 1/450 | 1/750 |
| --- | --- | --- | --- | --- | --- |
| Take this much plasma | 27 uL | 19 uL | 15 uL | 12 uL | 11 uL |
| Add this much DCC | 54 uL | 38 uL | 30 uL | 24 uL | 22 uL |
|  | Vortex, let sit at room temperature for 30 min. | | | | |
|  | Spin | | | | |
| Take this much supernatant | 51 uL | 27 uL | 15 uL | 6 uL | 6 uL |
| Add this much buffer | 255 uL | 273 uL | 315 uL | 294 uL | 494 uL |

Tips:

- Use 0.5 mL microvials for this step.

- Centrifuge at high enough speed for about 5 minutes so that the charcoal is completely pelleted.

B) Prepare ^3^H-CORT

We want to use enough hot CORT to saturate the CBG so that when we separate bound from free, every CBG molecule is providing a signal. However, the NSB signal becomes more prominent at higher concentrations, so it is a balancing act to find the ideal CORT concentration.

Moreover, because we expect variation in CBG levels, we want to add enough hot CORT to ensure that there isn’t ligand depletion in animals with high CBG levels.

One approach is to use the rule of thumb that a CORT concentration of 20X the Kd will saturate. This will typically be far along enough on the asymptote of the saturation binding curve to ensure good CBG saturation, but not so far along that NSB is an issue. However, for every new species, inspect the saturation binding curve and make sure that this approach makes sense.

20X the Kd is your final concentration. Make up your stock solution of ^3^H-CORT at 3X this concentration. For example,

- if your species has a Kd of 0.3 nM, you want a final concentration of 6 nM

- so, make up stock at 18 nM ^3^H-CORT.

Running 3TB and 2 NSB, you will need 5 tubes * 50 uL per sample = 250 uL hot CORT per sample, plus 150 uL for 3 QC tubes on every filter (harvester method) or 150 uL for 3 QC tubes in every run (charcoal method). For the harvester method, use this chart to calculate the volume of hot CORT to prepare:

| Number of Samples | 27 | 54 | 81 |
| --- | --- | --- | --- |
| Filters | 3 | 6 | 9 |
| Volume of hot CORT needed | 7200 uL | 14,400 uL | 21600 uL |
| Make this volume | 7500 uL | 14,700 uL | 22,000 uL |

Refer to the Hormone Calculator spreadsheet for figuring out how much hot CORT is required. Volume of hot CORT is based on the activity of the isotope batch and the desired 3X concentration of hot CORT.

Example: If you want a final hot CORT concentration of 20 nM, and you need 7.5 mL for your run, then you need to make up 7.5 mL of 60 nM hot CORT.

If your ^3^H-CORT batch is 92.9 Ci/mmol, then you need to add 41.8 uL of undiluted ^3^H-CORT stock to 7458 uL buffer.

NOTE: This uses a lot of hot CORT because we are using 100% hot hormone to nearly saturate the CBG in the sample. If you decide it is more economical for your volume of work, if it is possible to use a hot/cold mix (e.g. 50% hot).

C) Prepare Cold CORT in buffer for NSB tubes

We want 4 µM final concentration (this is a generic value; use 1000X K_d_ if the K_d_ is much greater than 4 nM), so make 12 µM stock.

The amount to prepare depends on how many runs, but you will need cold CORT for 2 NSB tubes * 50 uL per tube * x samples. Here is a chart for 12 uM stock:

| Number of Samples | 27 | 54 | 81 |
| --- | --- | --- | --- |
| Filters | 3 | 6 | 9 |
| Min. volume of cold CORT needed | 2700 uL | 5400 uL | 8100 uL |
| Take this much 1 mg/mL cold CORT in EtOH | 13 uL | 26 uL | 37 uL |
| Add to this much buffer | 2987 uL | 5974 uL | 8463 uL |
| Final volume | 3000 uL | 6000 uL | 8500 uL |

D) Set up assay

For the charcaol and harvester methods, put 50 µl of the ^3^H-CORT mix in each of 3 scintillation vials. These are your total counts tubes (TOTCNT).

For the charcoal and dialysis methods, set up in 12 x 75 test tubes in racks.

--each sample will have 3 total binding tubes (TB) and 2 non-specific binding (NSB) tubes.

--Add reagents as follows:

|  | TB tubes | NSB tubes |
| --- | --- | --- |
| 3X plasma | 50 uL | 50 uL |
| buffer | 50 uL |  |
| Cold CORT |  | 50 uL |
| 3X ^3^H-CORT | 50 uL | 50 uL |

Centrifuge briefly if needed to get droplets off the sides, then gently vortex.

Incubate overnight in fridge.

Add scintillant to TOTCNT vials, vortex, and set aside to count with assay

E) Prepare for separation (charcoal and harvester methods)

For harvester, soak filters in rinse buffer + 0.3% PEI in refrigerator (GF/B filters) for 1 hour prior to harvesting.

For charcoal, chill centrifuge and prepare dextran coated charcoal.

F) Separate and count (charcoal and harvester methods)

Follow protocols for separating bound from free hormone for DCC or harvester as appropriate (Section 3 below).

2.2 MCBC by Dialysis Method

A) Strip and Dilute Plasma

For the dialysis method, if using a 1/10 final plasma dilution, strip the plasma samples using 1:1 volumes of plasma and DCC. If using more dilute plasma, you can strip with 2 parts DCC to1 part plasma as with the charcoal and harvester methods. Unlike the other methods the dialysis protocol mixes plasma with an equal volume of hormone/buffer resulting in a 1/2 dilution. Therefore, plasma is made up here to 2X the final concentration.

One problem with the HTDialysis plates is that wells can leak, especially during long incubations. As a result, we typically prepare 4 TB wells and 2 NSB wells. Therefore, each plasma sample needs 6 wells x 75 uL of 2X plasma = 450 uL 2X diluted plasma. However, because we will be dividing the stripped plasma into separate vials for TB and NSB wells, prepare 600 uL of 2X stripped plasma.

E.g.: - take 135 uL raw plasma, add 135 uL DCC in 0.5 mL microtube; vortex, allow to sit 1 h.

- centrifuge and remove 240 uL supernatant into separate microtube

- add 360 uL buffer.

B) Prepare ^3^H-CORT

For the charcoal and harvester methods, the plasma is diluted enough that the remaining CBG will bind only a relatively small proportion of the hot CORT being added. For the dialysis method, plasma may be much more concentrated, meaning that it will bind a substantial amount of the hot CORT added (i.e., ligand depletion). This needs to be factored into the calculation of how much CORT to add.

The 20X Kd guideline for saturating CBG is meant to be free CORT. For example, if the Kd is 1.0 nM, you want 20 nM free CORT to saturate the CBG. If the plasma for dialysis is diluted to 1/10 and inspection of the saturation binding curve used to calculate the Kd suggests that the plasma for your species has about 30 nM CBG binding, then at 1/10 dilution the plasma will have 3.0 nM binding. Thus, to ensure that you have 20 nM free CORT, you will need to add 20 nM + 3.0 nM, or about 23 nM hot CORT. On top of this, we can expect that CBG concentration will vary among individual plasma samples. Thus, if the plasma pool used for the Kd saturation binding curve has less CBG than some individual samples, you will need even more hot CORT to ensure that these high-CBG samples are fully saturated. Exactly how much extra CORT needs to be added will depend on the variability in CBG. This can only be determined by a trial MCBC run, inspecting the results, and deciding whether there is a risk that you aren’t saturating the CBG. For example, if you find that some samples have as much as 70 nM CBG binding, you would be advised to increase the hot CORT to 27 nM.

NOTE: This uses a lot of hot CORT if using 100% hot hormone. It is almost certainly more economical in the dialysis method to use a hot/cold mix because you will be using more concentrated plasma and higher CORT concentrations. We often use 10% hot CORT for dialysis.

Unlike the charcoal and harvester methods, it is not advisable to add multiple 50 uL volumes to the dialysis wells. Air bubbles get introduced and it is not possible to vortex the plate enough to mix solutions together. We therefore prepare the mixtures before pipetting into the dialysis unit. This also means that – as with the plasma – we make up CORT to 2X concentrations, not 3X as for the other seaparation methods. The procedure can be a bit confusing at first, but the following box illustrates how we do this (Note that the Box uses 20% plasma in the example; we now typically start with 10% plasma):


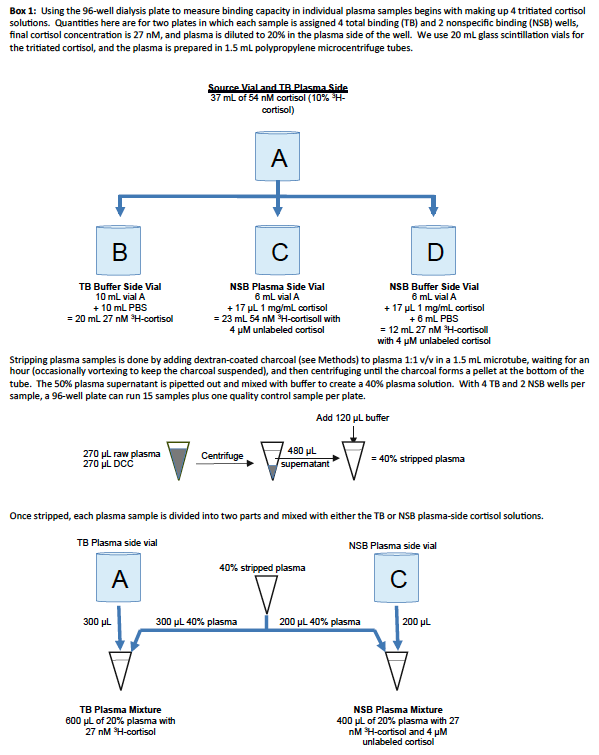


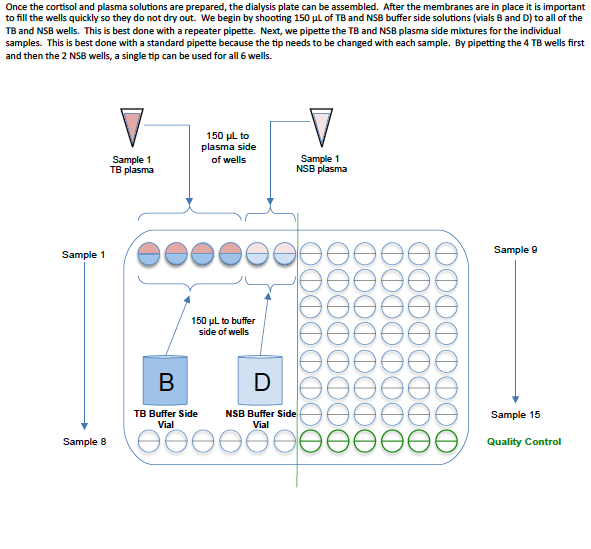


C) Prepare and Pipette into the Plate

Once the CORT vials and plasma mixtures have been made up, it is time to prepare the membranes and assemble the dialysis plate.

We use Spectra/Por 2.5 cm wide cellulose dialysis tubing with M.W. cutoff of 12,000-14,000. Using rubber gloves and a razor blade, we cut the tube to a length that fits between the posts of the dialysis unit (about 11.5 cm). Then we slice off two thin strips from the long edges of the tubing so that we will have two strips that can be separated once the dry membrane is hydrated and washed.

To hydrate the membrane strips, place them in distilled water or buffer for 1 h. Then transfer to a 20% ethanol mix made with buffer or water, and soak for another 20 minutes. They can be kept in the 20% ethanol for weeks (we use water instead of PBS if we are storing for weeks). Before use, rinse twice in PBS.

To assemble the unit, follow the directions provided by HTDialysis. Remember that once the membranes are removed from PBS, they MUST NOT dry out or holes will form. Therefore, once assembled, invert the unit and bang it down over paper towels to remove excess buffer. Then, quickly pipette the buffer side TB and then the buffer side NSB solutions using repeater pipettors. This will keep the membranes hydrated as you add the plasma side solutions. Cover with cling film or adhesive plate covers.

C) Equilibration Times

Vortex the plate (use the multi-tube vortexer), being sure not to slosh the contents too much. Place the plate in the fridge to equilibrate. If possible, keep the plate on a plate shaker of some description while incubating. Otherwise, periodically remove the plate and vortex gently.

By placing CORT on both sides of the wells and by using high plasma concentrations, we minimize equilibration time. Even at 4C, we have found that samples can take as little as 2 hours to equilibrate. It is critical to test the equilibration time for your particular samples, because ending prematurely will underestimate binding.

In theory one could leave plates overnight to equilibrate. However, we have found that the wells have a tendency to leak over time, so leaving the plates for too long will result in the loss of data. Therefore, test the equilibration time and use as short a period as will reliably ensure good measurements.

D) Measuring Binding

One the equilibration period is done, remove the plate from the fridge. Acting as quickly as possible (to avoid the unit warming too much during sampling), pipette out 100 uL from the plasma and buffer sides of each well into separate scintillation tubes.

Obviously, it is critical that you are able to match the buffer side and plasma side tubes for each individual well, and that you know what wells are the TB and NSB wells for what sample. Organization and consistency are essential.

Once the wells are all pipetted into scintillation vials, add 3 mL of scintillation fluid (any type compatible with aqueous samples is fine, but be sure to use the same scintillation fluid for every run as the counting efficiency will vary between brands).

Count the samples and enter the data into the Dialysis MCBC spreadsheet (Supporting File 5). Free CORT can be calculated once you enter the total CORT and Kd value (at body temperature) for your species.

3. Separation Methods

Separation of bound and free hormone is essential for any measurement of CBG. This separation can be done with dextran-coated charcoal (DCC), with the use of a cell harvester, or with a dialysis plate. This document provides basic protocols for all three methods.

The methods use different buffers, and the nature of the buffer is critical. For the DCC and dialysis methods, “buffer” always refers to PBS with 0.1% gelatin. For the harvester, the rinse buffer used in the separation is 25 mM Tris HCl (which is different from the assay buffer used in the incubations, which is 50 mM Tris acetate). In both cases, pH to 7.4. For Tris buffers, the pH must be done at the incubation temperature (4°C or 37°C).

A. Dextran-coated Charcoal Separation

Background:

Dextran-coated charcoal adsorbs free steroids, but not (for the most part) CBG-bound steroids. However, as free hormone is adsorbed, the amount of CBG-bound hormone will tend to decrease in order to reach a new equilibrium of bound:free. To slow the rate of loss of CORT from CBG, we do the DCC separation at 0°C. We also try to minimize the length of time between when DCC is added and when we centrifuge it down (the “DCC exposure time”). The DCC exposure time is experimentally determined. Too short and the charcoal doesn’t have time to grab all the free hormone. Too long and the charcoal starts stealing too much hormone from CBG. We have determined that 15 minutes DCC exposure is ideal. However, a significant amount of bound CORT is lost to CBG over this time. This loss of bound CORT must be accounted for in order to estimate the maximum binding capacity. The loss of bound CORT during DCC exposure is calculated by the “Charcoal Adjustment Protocol” and it must be done for every new species prior to running the MCBC assay, and for most consistent results, it should be done for every new batch of DCC because the rate of adsorption seems to change slightly but significantly between batches.

Important Notes:

The current saturation binding and MCBC assay procedures have a reaction volume of 150 µL. Here we will add 300 µL DCC to do the separation. After centrifuging, we decant the entire supernatant into the scintillation tube. If the decanting method doesn’t work (i.e. if the charcoal pellet crumbles too easily and comes out with the supernatant), then you will need to pipette out a volume of the supernatant (e.g. 300 or 350 µL of the supernatant), meaning that you will only counting a fraction of the actual binding. Calculating actual maximum binding capacity requires adjusting for this. If you pipette supernatant instead of decanting, the volume of supernatant that you add to the scintillation vial must be reflected in the MCBC spreadsheet!

Separation Procedures:

1. Turn on the centrifuge and turn to 0°C. Get a manual repeater pipettor with a 10 mL tip (electronic repeaters are not fast enough). The very end of the tip should be cut off so that it is not clogged by the larger charcoal particles. Set the pipettor to dispense 300 µL per shot.

2. In a beaker with a magnetic stirrer, prepare as much DCC as you will need (with about 100 mL excess to ensure easy pipetting).

3. Prepare an ice slurry (as near as possible to 0°C) for the tube racks and another for the DCC beaker.

4. Place the tube racks with your samples in the slurry.

5. Set a timer for 16 minutes, start the countdown and pipette 300 µL DCC into the tubes within 1 minute. This is possible if you pipette very quickly – practice before pipetting a full run of real data.

6. Transfer the tubes to centrifuge buckets, balancing buckets within ~ 0.1 g.

7. Once the timer ends, the DCC exposure is done. Spin the tubes at 2500×g for 12 minutes.

8. Decant the supernatant into scintillation tubes, then add 2.5 mL scintillation fluid.

9. Vortex thoroughly and allow to rest for about 2-4 hours prior to reading in scintillation counter. Enter the data into the MCBC spreadsheet (Supporting File 4).

B. Harvester Separation Method

(48 place harvester)

Background:

The harvester captures CBG in the filter (Whatman GF/B glass fiber filters) but lets free hormone pass through. The polyethylenimine (PEI) coating of the filter is absolutely essential to the capture of CBG. PEI is a polycationic polymer that binds to the negatively charged glass fibres and leaves a net positive charge. Much of what you read about PEI is that it reduces nonspecific binding to the filter. However, for CBG assays, its main function is actually to capture CBG.

The proper and consistent soaking of filters is therefore critical. This means using fresh PEI to ensure that there is enough PEI to coat the filters. In one assay day, I soak up to 4 filters in about 700 mL of soaking buffer, and then dispose of the rest at the end of the day (PEI waste does not go down the drain – it must go to chemical waste). Also, soaking the filter too long can result in a softening of the filter itself; I find that soaking filters for 45 minutes is sufficient to ensure good PEI coating. Once the filter has been in for much more than 90 minutes, it starts to get soft and is harder to handle.

Even with these precautions, the harvester tends to suffer from high inter-filter variation in capturing CBG and it is critical to have quality control samples that run on every filter.

In addition to the equipment that comes with the harvester, one needs a small plastic tub about that will just fit the probes. This is used to for receiving rinse buffer when running it through the lines and also for rinsing out the probes during cleanup.

Finally, before starting an assay, measure 3 mL of water into a test tube and place it in the test tube rack. Mark the 3 mL level on the corner of the rack so that you can see how much buffer to add during the assay.

Setup

1. Empty the buffer reservoir (this will just be dH_2_O from the last time the Harvester was used).

1. Fill the buffer reservoir with at least 2L (depending on the size of your assay) of refrigerated rinse buffer (25mM Tris HCl). Make sure there are no bubbles in the line.

Assuming 3 x 3 mL washes, each run will need 432 mL rinse buffer, plus surplus for lines etc., so estimate 500 mL, plus an initial 500 mL for flushing the lines. Thus, 2 L could be expected to last for 2-3 runs.

1. Place the buffer reservoir into a cooler filled with an ice slurry, making sure the slurry level matches the initial buffer level. This will chill the buffer to near 2°C.
2. Turn the solenoid switch (on the black box under the harvester platform) to “on”

# Filtration

1. Just prior to filtration, put on gloves.

1. Close the latches to secure the harvester head closed.
2. Make sure the Harvester handle is in “off” position. Turn on the vacuum pump.
3. Run cold buffer through the lines (3-5 sec) to make sure the lines are cold. This can be run into the small waste tub.
4. Turn the harvester handle to “harvest” to draw the cold buffer in the tub through the harvester to cool off the whole system. If you have more than one waste collection bottle, this can be drawn into the non-radioactive waste bottle; switch to the radioactive one for the next steps.
5. Turn the Harvester handle to the “off” position, open the latches and tilt back the harvester head. Align a pre-soaked filter on the supports surrounding the filtration area. Be careful not to touch the filter, except around the edges.
6. Turn the harvester handle back to the “harvest” position. This will pull a vacuum across the filter. Return to “off.”
7. Close the Harvester head and secure latches.
8. Place 500 mL distilled water in the small tub, lower the probes into the tub and move lever to “harvest.” This removes excess PEI (unattached to the filter) from the filter. Return to “off”
9. Get the first rack of tubes and place on the harvester platform. (Even if your assay does not require 48 tubes, you must have 48 tubes or buffer will empty onto the rack and counter).
10. Lower the Harvester probes into the test tubes and quickly suck up the incubate by moving handle to “harvest”. Follow immediately with rinse buffer: hold the probe tips just above the 3 mL rinse level that you marked on the rack. Press black button on probe unit to inject buffer, then release slightly before buffer reaches the 3 mL marking (buffer continues to flow for momentarily after you’ve stopped pressing the button).
11. Quickly lower probe assembly to suck up tube volumes second time. For a CBG assay, this suck/rinse process should be done 3 times for a total rinse volume of 9 mL.

1. Move the Harvester handle to the “off” position, turn off the vacuum pump.
2. Unlatch the harvester head and remove the filter from the harvester using flat forceps. If the run requires multiple filters, place filters in designated areas on sheets of plastic wrap a bit larger than the filter paper. Run all filters prior to transferring filters to scintillation vials.

NOTE: Be sure to check the liquid level in the waste jug after processing each filter. The vacuum pump will be damaged (and contaminated with tritium) if the liquid level gets too high and goes up the vacuum hose.

# Preparation of filters for counting

1. The rubber o-rings on the harvester head effectively score the filter. The filter “disks” can be removed with forceps. Remove the disks, being sure to keep the forceps at the outside edge of the disks.
2. Place the filter disks into a scintillation vial (we use 7 mL polypropylene vials). Try to stuff the filter disk right to the bottom of the vial.
3. Add 300 µl of 95-100% ethanol onto filters in each vial using repeater pipette. Place the rack of vials on a shaker and shake at low speed for 1 hour. (This critical step helps to free the ^3^H-CORT from the filter so that accurate counts are obtained).
4. Add 3 ml scintillation fluid (we use Optima Gold MV) to all vials, including any total count vials required by your assay.

1. Cap vials and vortex filters for at least 7 seconds (make sure filter paper is moving around in vial when vortexing), then shake for 1 h at 300 rpm on shaking platform (e.g. in the scintillation racks, strapped with velcro strap to the shaker).

1. Vials can be read immediately after the hour of shaking. If 1 h shake is not available, let sit for >12 hours before counting.

# Harvester cleanup

1. As soon as filtering is finished, place probe assembly in the small tub and spray the probes with 70% ethanol to remove radioactivity (this will take about 400 mL).
2. Add some distilled water (about 300 mL) and close and latch the harvester head. Suck up the water/alcohol from the small tub. Empty the waste collection bottle into radioactive waste liquid waste container located in the radioactive waste cabinet. If you don’t have two waste bottles, rinse out the waste bottle with 2-3 washes of Radiac and empty each time into the radioactive waste container. After that, the waste bottle should be “cold.”
3. Pour the remaining buffer in the rinse bottle back into the buffer stock bottle for later use.
4. Fill the buffer reservoir with at least 2L dH_2_O. Run through the probes (harvester in “off” position) into the small tub. Watch the level of water in the rinse reservoir carefully, so that you do not introduce air into the system.
5. Empty the tub, and refill with dH_2_O. Repeat for a total rinse of about 6 L of water.

1. Use some 70% ethanol to wipe down the platform and o-rings. Immediately rinse with dH_2_O so the o-rings are not dried out by the ethanol.
2. Turn off the solenoid.
3. Leave Harvester closed but unlatched. If it is left latched, the o-rings will become deformed. System should be left in “harvest” position.
4. Once a month, if the Harvester is getting regular use, run a 10% bleach solution through the system to inhibit algal growth. Rinse with several liters of dH_2_O.

C. Dialysis Separation Method

(96 well HTDialysis plate)

Background:

The HTDialysis plate is a reusable 96 well plate with vertical dialysis membranes through each well. The plate is open at the top, so reagents can be added and removed by simple pipetting rather than needing Hamilton syringes as required by other microdialysis techniques.

Each well receives a plasma mixture (typically 10% plasma) on one side and a buffer (PBS with 0.1% gelatin) mixture on the other side. Both sides have the same amount of hormone initially, in an amount sufficient to saturate the CBG. The CBG in the plasma side will bind some portion of the hormone, and the remaining free hormone will reach and equilibrium concentration on both sides of the membrane. To measure nonspecific binding (i.e. binding by albumin), some wells also have a huge excess of cold GC added (4 uM or greater).

After the setup reaches equilibrium, the plasma side and the buffer side of each well is read in a scintillation counter. The difference in the counts is a measure of the total (CBG+albumin) binding on the plasma side. In the NSB wells, the difference is just the albumin binding. Thus, the total binding less the albumin binding equals the specific binding by CBG.

The great advantage of the dialysis method is that the separation occurs as part of the process of reaching equilibrium. So, whereas DCC has a lengthy charcoal exposure time during which bound hormone comes off CBG and is adsorbed by the charcoal, in dialysis the plasma and buffer sides of the wells are sampled instantaneously. And whereas the harvester relies on the PEI coating on the glass filters to reliably capture CBG molecules, the dialysis membrane is physically preventing the movement of CBG so we can be certain that we are measuring all of the CBG.

Setup

There is no independent separation procedure for the dialysis method because the separation is inherent in the setup in the dialysis plate. See either the saturation binding protocol or the point sample (MCBC) protocol for dialysis plate (Section 2.2, above).

4. Charcoal Adsorption Assay

The DCC method of calculating MCBC involves a 10-15 minute exposure of the plasma/CORT mixture to DCC. During this time, we expect some bound hormone to come off the CBG and be adsorbed by charcoal. This effect is strengthened by the fact that adding the DCC increases the reaction volume, which will shift the equilibrium towards less binding. We try to counteract this loss of measured binding by doing the DCC separation at 0°C. However, we know that we cannot completely prevent the loss of bound hormone. Therefore, with this protocol we measure how much hormone we lose over the course of the DCC exposure.

A) Introductory Notes

- This assay is run for each species, and preferably for each batch of DCC used if doing a big run with multiple batches of DCC.

- It is run after the optimal plasma dilution and with the CORT concentration used fo rthe MCBC assay.

- Our experience is that it takes 10-15 minutes for the DCC to clear free hormone. That is, even in the absence of CBG (i.e. plain buffer), DCC needs at least 10 minutes to work. Thus, in this assay, the minimum exposure time is 5 min.

- Other work indicated that that beyond 60 minutes, most of the hormone has been removed and the loss of bound hormone slows considerably.

- DCC exposure times will be 5, 10, 20, 30, 45, and 60 minutes

1. Strip Plasma

--This assay needs 1650 uL plasma, so prepare at least 1900 uL.

--If using pre-stripped plasma, dilute to the optimal level as determined by the dilution protocol.

--If using unstripped plasma, do the initial 1/3 dilution with DCC in buffer according to the table below. If the necessary dilution is not in the table, use the hormone calculator spreadsheet to figure out the volumes.

| Final Dilution | 1/54 | 1/100 | 1/198 | 1/450 | 1/750 |
| --- | --- | --- | --- | --- | --- |
| Take this much plasma | 130 uL | 80 uL | 50 uL | 33 uL | 28 uL |
| Add this much DCC | 260 uL | 160 uL | 100 uL | 66 uL | 56 uL |
|  | Vortex, let sit at room temperature for 30 min. | | | | |
|  | Spin | | | | |
| Take this much supernatant | 330 uL | 180 uL | 90 uL | 39 uL | 24 uL |
| Add this much buffer | 1650 uL | 1820 uL | 1890 uL | 1911 uL | 1976 uL |

1. Prepare ^3^H-CORT

--As usual, this is prepared at 3X final concentration.

-- For 3 TB and 2 NSB per time period, you will need 5 tubes * 50 uL per tube * 6 time periods = 1500 uL hot CORT per run, plus 3*50 for TOTCNT, so make 1900 uL

--Use the Hormone Calculator spreadsheet to make up 1900 uL of 3X hot CORT.

Example: If K_d_ = 0.3, then you want a final CORT concentration of 6 nM. Therefore, make up 1900 uL of 18 nM hot CORT.

--add 50 uL of the 3X hot mix to three TOTCNT scintillation tubes and set aside.

1. Cold CORT

--There are 6 times with 2 NSB tube, so you need 600 uL cold CORT. Make 1000uL.

--want 4 µM final concentration (this is a generic value; use 1000X K_d_ if the K_d_ is much greater than 4 nM), so make 12 µM stock.

-- Take 43uL of 0.1 mg/mL cold CORT, add 957uL buffer.

4. Set up assay

--Set up in 12 x 75 test tubes in racks. Turn on the centrifuge to get it to 0°C.

--Each DCC exposure time will have 3 total binding tubes (TB) and 2 non-specific binding (NSB) tubes.

--To the TB Tubes: add 50 uL buffer, 50 uL diluted plasma, and 50 uL hot CORT.

--To the NSB tubes: add 50 uL cold CORT, 50 uL diluted plasma, and 50 uL hot CORT

--To 3 scintillation vials, add 50 uL hot CORT

--vortex each test tube (centrifuge briefly if needed to get droplets off the sides)

--incubate for at least 4 h in the fridge, preferably overnight.

--Prepare the DCC for separating. Do all separations at 0°C.

--Start a 60 min timer. Take the 60 min tubes, add DCC (300 uL) and keep in the ice slurry.

--Wait 15 minutes and add DCC to the 45 min. tubes, continue this way for the rest of the tubes

--Load all tubes into the chilled centrifuge.

--When the timer goes off, spin.

--decant the supernatant into scintillation vials.

--add scintillant to all tubes, including the TOTCNT tubes, vortex, and count.

--enter data into the Charcoal Adjustment spreadsheet (Supporting File 6). This will let you visualize how long you should let the DCC sit before centrifuging during the MCBC assay, and then you can calculate the adjustment factor to be applied for that exposure time.

--NOTE: some species, especially birds, lose bound hormone very quickly (50% of bound hormone or more). If your species has this problem, you may want to rething using the DCC method. The faster the loss of bound hormone to the charcoal, the more error you will have due to slight changes in the length of DCC exposure between runs and from the first sample to the last sample.

Appendix: Reagent Recipes

Recipes for Dextran-coated Charcoal (DCC) Assays

Phosphate buffered saline with gelatin is susceptible to bacterial and fungal growth which will interfere with the assays. The PBS recipe here uses thimerosal to inhibit such growth, but it is nonetheless recommended to use PBS within a month even with thimerosal, and within a week if no thimerosal is used.

DCC:

Ingredients:

6.25 g Activated Charcoal (Sigma Activated C5260; Norit A)

0.625 g Dextran 70 (Sigma 31390)

Ultrapure water

Directions:

- Dissolve dextran in 100 ml water in a 250 ml graduated cylinder (heating to 40ºC can speed this process)

- Add the charcoal and mix well.

- Settle for about 4 hours and decant any fines, keeping charcoal residue and refilling with water.

- Repeat several times.

- After decanting the last time, make up to a final volume of 100 ml (with water).

Store at 4ºC.

For assays make up a 1/10 dilution of this charcoal concentrate with assay buffer (i.e. add 9 mL buffer for each1 mL concentrate).

Alternative DCC Recipe

Whereas the previous recipe uses a charcoal concentrate that is made up with water and can therefore be stored for months in the fridge, this recipe needs to be made fresh, since it is made with PBS. We have used both recipes with good results.

For 200 mL DCC:

Dissolve 0.1 g dextran 70 (Sigma, 31390) in 200 mL PBS in beaker with magnetic stirrer. (about 20 min)

Add 1.0 g of activated charcoal and stir for another 20 minutes.

The DCC is ready to use.

Phosphate Buffered Saline (PBS) with 0.1% Gelatin

8.66 g Na_2_HPO_4_ = sodium phosphate dibasic (anhydrous)

6.40 g NaH_2_PO_4_ ^.^ 2H_2_O = Sodium phosphate monobasic dihydrate

0.1 g Thimerosal ** NOTE: ** Dangerous – absorbs through skin

9.0 g NaCl

1.0 g gelatin

To dissolve take 800 mL of ultrapure water in 1000 mL beaker, add the chemicals, and place a magnetic stirring rod in the beaker. Place beaker on a stirring hot plate (set to about 80ºC) and cover beaker with plastic wrap. Stir until the gelatin and chemicals are all dissolved. NOTE: gelatin takes a long time to dissolve fully – hold the beaker up to the light and look for “specks of diffracted light”; when gelatin is fully dissolved, these specks will disappear.)

Cool to room temperature.

Transfer to a volumetric flask (keep beaker for next step) and top up with ultrapure water to 1000 mL

Transfer back to the beaker, drop in a magnetic stirrer and adjust to a pH of 7.4 by adding NaOH as required.

Store in fridge.

NOTE: Consider omitting the thimerosal and autoclaving buffer instead. Even with thimerosal, RIA buffer should preferably be used in a week and not be kept more than 4 weeks due to the risk of fungal or bacterial growth. Making 2 or 4 L of buffer and autoclaving buffer in 500 mL or 1 L bottles could be more efficient.

Recipes for Harvester-based Assays

Tris buffers are used for the harvester method. Tris can be stored at room temperature or in the fridge for moths without any bacterial or fungal growth.

Assay Buffer (50 mM Tris Acetate)

Tris buffers need to be pH’d at the intended assay temperature because pH changes dramatically with temperature. Most assays will be done at 4^o^C, but saturation binding curves done at 37^o^C will require assay buffer with the pH set at 37^o^C.

| Water | 1 L | 2 L |
| --- | --- | --- |
| Trizma base | 6.05 g | 12.11 g |

Chill to 4^o^C, then pH to 7.4 with glacial acetic acid.

(or warm to 37^o^C and pH with glacial acetic acid if running the intended assay at 37^o^C)

NOTE: Using 5N acetic acid instead of glacial makes it possible to do outside of a fume hood

Rinse Buffer (25 mM Tris HCl)

| Water | 2 L |
| --- | --- |
| Trizma base | 6.05 g |

Chill to 4^o^C, then pH to 7.4 with concentrated HCl (we use 6N).

NOTE 1: It is critical to use HCl and not Acetic acid for the rinse buffer. Acetic acid appears to interfere with specific binding by the filter.

NOTE 2: Even for assays run at 37^o^C, the rinse step is done at 4^o^C, therefore all rinse buffer should be pH’d at 4^o^C.

Rinse Buffer + 0.3 % PEI (for soaking filters)

| 25 mM Tris rinse buffer | 1 L |
| --- | --- |
| Polyethylenimine (PEI) | 6 g |

PEI (Sigma P-3143) is an extremely viscous liquid that is 50% PEI w/v with water. Because it is half water, a 0.3% solution requires about 6g/L rather than 3g/L. I use the following procedure to make 1 L of the soaking buffer:

1. Weigh an empty 50 mL falcon tube; record the weight. In the fume hood, add what looks like about 5 mL of PEI.

2. Weigh the tube again and calculate the mass of PEI. Add more PEI as needed to have in the range of 6-7 g of PEI. Calculate the volume of buffer required. For example, if you have 7.3g PEI, you need 6g/1L = 7.3 g/xL; x = 7.3/6 = 1.22 L

3. Add about 30 mL of 25 mM Tris to the falcon tube and let it sit for several hours or overnight. Ideally, place the falcon tube on a shaker or in a water bath. After sitting for several hours, the PEI will be easier to dissolve into the buffer.

4. Shake the falcon tube well, add to a 2L bottle. Add several more rinses of 25 mM buffer to the falcon tube to completely get all the PEI, emptying each time into the 2L bottle. Add 25 mM buffer to the bottle to bring it up to 1L, then add the extra 220 mL with a graduated cylinder.
